# Side chain flexibility and the symmetry of protein homodimers

**DOI:** 10.1371/journal.pone.0235863

**Published:** 2020-07-24

**Authors:** Yaffa Shalit, Inbal Tuvi-Arad

**Affiliations:** Department of Natural Sciences, The Open University of Israel, Raanana, Israel; University of Leeds, UNITED KINGDOM

## Abstract

A comprehensive analysis of crystallographic data of 565 high-resolution protein homodimers comprised of over 250,000 residues suggests that amino acids form two groups that differ in their tendency to distort or symmetrize the structure of protein homodimers. Residues of the first group tend to distort the protein homodimer and generally have long or polar side chains. These include: Lys, Gln, Glu, Arg, Asn, Met, Ser, Thr and Asp. Residues of the second group contribute to protein symmetry and are generally characterized by short or aromatic side chains. These include: Ile, Pro, His, Val, Cys, Leu, Trp, Tyr, Phe, Ala and Gly. The distributions of the continuous symmetry measures of the proteins and the continuous chirality measures of their building blocks highlight the role of side chain geometry and the interplay between entropy and symmetry in dictating the conformational flexibility of proteins.

## Introduction

Symmetry plays a central role in protein structure [1–6]. In the past two decades, various researchers have suggested that symmetry is associated with increased structural stability, higher efficiency of oligomerization mechanisms, possible reduction of errors in biological synthesis and allosteric regulation, among others [1–4, 7]. Nevertheless, the majority of protein clusters do not reach perfect symmetry [8]. Imperfect symmetry in clusters–even those built by identical units–is a key phenomenon which, to the best of our knowledge, still awaits detailed quantification and exploration of its structural origins.

Over the years, efforts have been made to define and quantify protein symmetry levels using various methods based on quaternary-structure-alignment algorithms [9–19]. These methods involve superposing one peptide on another, and estimating their alignment by root mean square deviation (RMSD) of matching α-carbons, or by a related scoring formula. Such calculations often take into account only part of the protein atoms [18–21]. Pednekar and Durani [22] showed that the cause for symmetry breaking of homomers is the aliphatic side chains of amino acids, whereas amino acids with aromatic side chains preserve their symmetry. Swapna et al. [23] calculated the global and local symmetry of homodimers using the GloA_Sc measure, which is based on C_α_-C_α_ distances, as originally proposed by Andre et al. [3] They found that most homodimers are highly symmetric and only about 3% of them can be considered as asymmetric based on this measure. According to their study, the conformational differences between the chains and the differences in their orientation contributed to the global asymmetry. They concluded that the asymmetry of these homodimers is spread over the entire protein structure.

The continuous symmetry measure (CSM) method [24–26] has been recently proven useful in quantitatively describing the near-symmetry of protein homomers. Bonjack-Shterengartz and Avnir showed that the main distortion often originates in amino acids located near the symmetry axis and in the border regions of the clustered oligomers. They also found that hydrophilic amino acids are more likely to carry conformational-symmetry distortions [27, 28]. In another study, they used the CSM method to locate the hinge region of domain-swapped protein dimers, which is considerably less symmetric than the rest of the protein dimer [29].

Another recent application of the CSM to protein structure is the addition of residue chirality as a third dimension of Ramachandran plots [30, 31]. This addition has revealed hidden conformational information that improves the understanding and characterization of protein structure. The method can help identify the location of special joint points along the protein sequence where a change in direction occurs, such as α-helix kinks and β-strand twists, as well as points at which the secondary structure changes [31]. The CSM method is designed to find the minimum normalized distance between an examined molecule and the nearest structure with the desired symmetry. The calculation can be based on the complete set of atoms, the protein backbone, or any other relevant molecular fragment [27]. Unlike other methods, a CSM calculation of the whole molecule takes into account the full structure of the examined molecule rather than the C_α_ or backbone atoms only. It therefore offers a way to examine quantitatively the contribution of the side chains to the overall symmetry.

In this study, we used the CSM methodology to investigate the symmetry level of protein homodimers. We analyzed two aspects of the coordinates of 565 proteins from X-ray crystallographic measurements extracted from the RCSB-PDB website [32, 33]: the rotational symmetry of the complete protein structure, and the conformational similarity of equivalent residue pairs. Our results suggest that the overall symmetry level of the proteins deviates from their backbone symmetry level. Furthermore, the conformational flexibility of the side chains, as quantified by their continuous chirality measure, shows that the source of this deviation is a small group of amino acids whose tendency to create local distortions is higher than that of the other amino acids. The results also indicate that amino acids form two groups: distorting residues and symmetrizing residues.

## Methods

### Continuous symmetry and chirality measures

The CSM represents the minimal distance of a molecular structure from a structure of the same set of atoms and bonds that belongs to the point group *G*. The measure represents symmetry on a continuous scale, [0,100], where 0 represents perfect symmetry of the original structure and 100 is obtained in the extreme case where the nearest symmetric structure collapses into the center of mass. The method uses the original structure as a starting point, and systematically searches for perfectly symmetrical structures with the same connectivity map. It then chooses the one which is closest to the original structure, according to the formula:

$$S(G)=100\cdot \frac{\min\left[\sum_{k=1}^{N}{\left|Q_{k}-P_{k}\right|}^{2}\right]}{\sum_{k=1}^{N}{\left|Q_{k}-Q_{0}\right|}^{2}}$$ (1)

Here {**Q**_*k*_} is the set of coordinates of the original structure atoms, {**P**_*k*_} is the set of coordinates of the symmetric structure atoms, and *N* is the number of atoms. The denominator is a normalization factor, given by the sum of the square distances of each of the original structure atoms from the center of mass, **Q**_0_.

The main challenge in CSM calculation is finding the closest symmetric structure. An exact algorithm has been recently developed for small- to medium-sized molecules [34], in which all structure-preserving permutations are scanned to find the closest symmetric structure. In the case of proteins, this algorithm is not applicable due to the enormous number of possible permutations, which requires an approximate calculation. For this purpose, a greedy algorithm was developed in 2011 [24], but it did not take into consideration peptide permutation. As a result, it often failed to find a permutation that preserved the protein sequence and the peptide structure unless such a permutation was defined *a priori*. Recently, our group has developed an improved approximated solution to the problem by means of an algorithm that overcomes these obstacles and is able to calculate symmetry measures of proteins within a reasonable time and with significantly higher accuracy and reliability [35]. This algorithm uses the Hungarian algorithm [36] to efficiently solve the assignment problem and find the correct permutation. It utilizes the sequence of the peptides to reduce the size of equivalence groups of atoms, and compels the code to preserve both the sequence and the peptide structure.

To evaluate the symmetry of protein homodimers, we retrieved the coordinates of 565 high-quality protein homodimers from the RCSB-PDB website (see below) and calculated for each the deviation from *C*_2_ symmetry, *S*(*C*_2_), both for the full set of atoms and at the backbone level (ignoring the side chains of the residues). This gave us an overall picture of the level of symmetry in our data set. To explore the sources of symmetry imperfections, we also used the continuous chirality measure (CCM) [37] as a similarity parameter to estimate the conformational differences between sets of corresponding residues. The CCM is derived from the CSM by minimizing Eq (1) over the chiral point groups (*S*_n_):

$$CCM=\min\left[S\left(S_{n}\right)\right]\;n=1,2,4,6,\ldots$$ (2)

Like the CSM, the CCM is a global parameter of the coordinates with a continuous scale in the range [0,100], with 0 representing an achiral structure (e.g., planar). As the measure grows, the structure becomes increasingly chiral.

**Fig 1:**
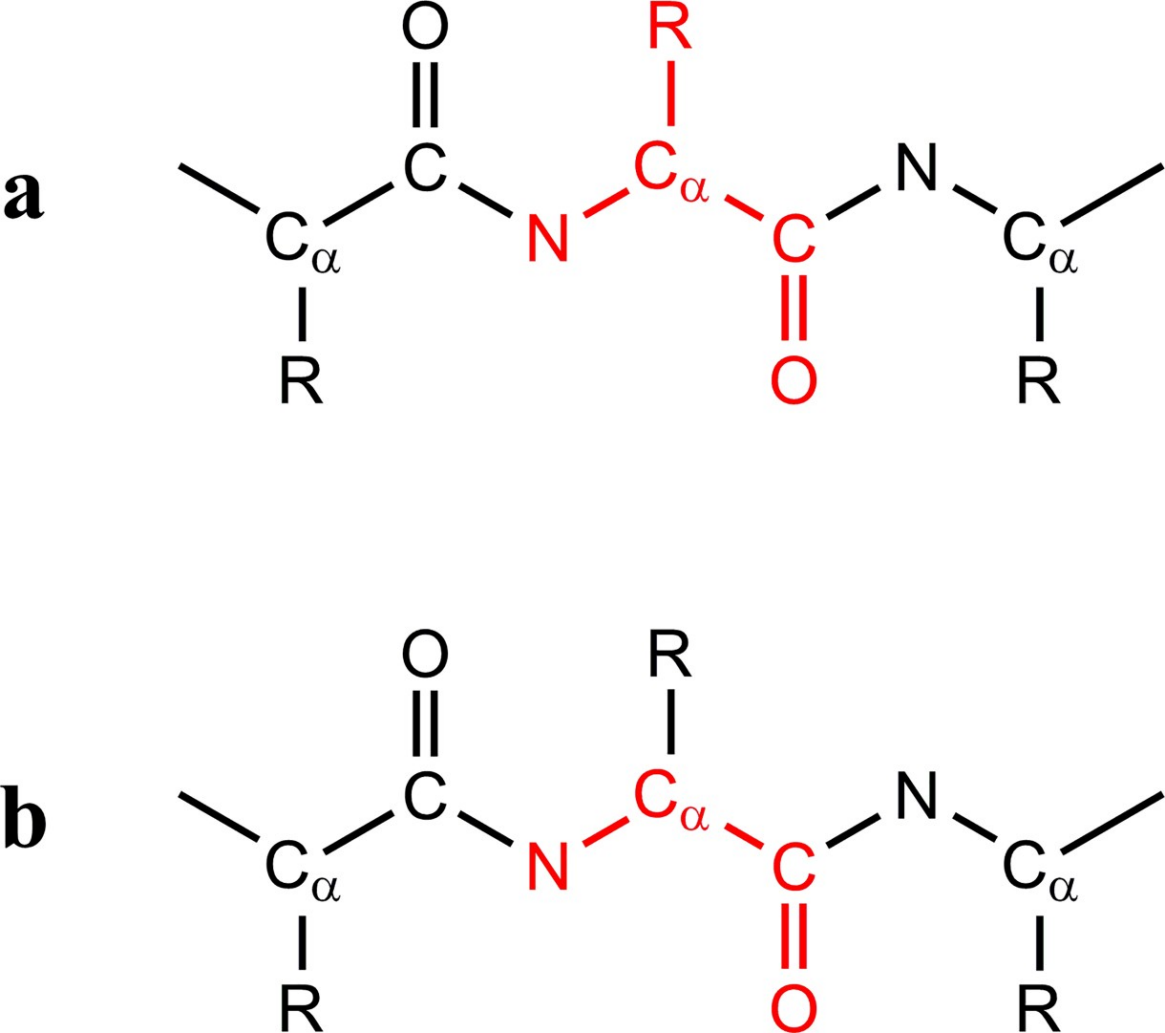
Subunits used for CCM calculations. a. The complete subunit (marked in red), comprised of all of the atoms of the relevant amino acid. b. The backbone subunit (marked in red), comprised of the atoms (N, C_α_, C, O) of the relevant amino acid. Hydrogen atoms were excluded in both cases.

To calculate the chirality of protein residues, a subunit had to be defined. To this end, we used two definitions, presented in Fig 1. The first, referred to below as the "complete subunit" (Fig 1A), was comprised of all the atoms of the studied amino acid, including the side chains. The second, referred to as the "backbone subunit" (Fig 1B), was comprised of the backbone atoms of the studied subunit without the side chains, i.e., the atoms N, C_α_, C and O. Hydrogen atoms were excluded from both subunits since they are generally absent from X-ray crystallographic protein structures. To cut the proteins into subunits and calculate the CCM, we used our in-house-developed Perl program pdbslicer (see below).

A note of caution is in order here. To estimate the symmetry deviation of specific amino acids, one might choose to calculate *S*(*C*_2_) for a pair of matching residues. While this calculation has its advantages being a direct symmetry measure, a drawback arises upon comparing the symmetry of matching pairs of the same residue located in different positions in the protein (e.g., near or far from the protein rotation axis). Due to the normalization factor in Eq (1), residues that are farther apart from each other may yield a smaller measure than residues that are closer together, even if the conformations of the residues are exactly the same. The effect of this inherent characteristic of the measure itself may be small, but it is difficult to quantify. Calculating the CCM of each residue separately overcomes this obstacle as the distance of the pair of residues from the center of mass does not enter into the calculation. However, this gives rise to a different caveat—the CCM of two structures can be the same, even if their conformation is somewhat different. Nevertheless, it is clear that if the CCM values are different, the conformations would also be different.

### Data preparation

The data were prepared in several stages, using various utilities. The stages included selecting and filtering PDB files, cleaning extra or erroneous data, and cutting the proteins into subunits. All the procedures are described in detail below.

### Data selection

PDB files of crystallographically resolved homodimers were retrieved from the RCSB-PDB website [32, 33] using RCSB search tools (https://www.rcsb.org). The search criteria were: (a) X-ray resolution ≤ 1.50 Å; (b) homomers with *C*_2_ symmetry as defined by the RCSB-PDB website; (c) exactly two chains in the biological assembly and exactly two chains in the asymmetric unit; (d) DNA, RNA or hybrid chains were filtered out; (e) 70% sequence identity. The search yielded 848 PDB files with good resolution and a unique identity.

As a control group we used 150 proteins that matched criteria (a), (b), (d) and (e), had exactly two chains in the biological assembly and exactly four chains in the asymmetric unit (i.e., two dimers each, 300 dimers in total). The data files of each set were cleaned using our in-house tool, pdb_prep (described next). At the end of this process, we obtained 565 homodimers with one dimer in the asymmetric unit (the "main set"), and 80 homodimers with two dimers in the asymmetric unit (the "double-dimers set"). Note that there was no need to use averaged B-factors, representing the mean square isotropic displacement of each atom [38], as an additional filter since the listed filters reduced these values to less than or equal to 46 Å^2^ for the main set (17 Å^2^ on average) and 28 Å^2^ for the double-dimers set (17 Å^2^ on average).

### Data cleaning

Many PDB files contain inconsistencies in the form of missing residues or missing atoms, low-level R_free_ values as well as extra data that are not required for symmetry evaluation (e.g., ligands). Our in-house code pdb_prep was used to clean the files that we downloaded and prepare them for CSM and CCM analysis. For each protein, we calculated the R_free_ grade as defined by *FirstGlance in Jmol* [39], which measures the quality of fitting a simulated diffraction pattern to the analyzed experimental diffraction pattern to further filter the data set. Files were kept if their R_free_ grade was at least "average" at their resolution. Next, we discarded any hydrogen atoms, solvent atoms, ligands and ANISOU data. In the case of atoms with alternate locations, we kept the first set of coordinates only. Next we matched the two chain sequences utilizing remark 465 (missing residues) and remark 470 (missing atoms) of the PDB files. If one chain was found to be longer than the other, the extra atoms or residues were discarded. Finally, the code verified that at least one biological assembly could be constructed based on the given coordinates without symmetric multiplication (by means of remark 350), to avoid perfect symmetry by construction. Files that failed to meet this criterion were excluded. At the end of the preparation process, we had 565 clean homodimers with one dimer in the asymmetric unit, ready for analysis.

For the double-dimers set we used the information in remark 350 (generating the biomolecule) to divide the four peptides in each file into two homodimers, each with two different chains. If more than two biomolecules were defined we used the first two options. The first homodimer was included in set I and the second in set II. This process yielded two sets of 80 homodimers. PDB-IDs of all the proteins used in this study are listed in S1 Appendix.

### DSSP and RMSD

Secondary-structure data and Ramachandran dihedral angles of each residue were retrieved from the DSSP (Dictionary of Protein Secondary Structure) website (https://swift.cmbi.umcn.nl/gv/dssp/index.html) [40, 41]. RMSD values for each protein were determined using MOE [42]. For a homodimer, the RMSD measures the average distance between the atoms on one chain and the atoms on another superposed chain. Mathematically it is defined by:

$$RMSD=\min\left[\sqrt{\frac{\sum_{k=1}^{N}\delta_{k}{}^{2}}{N}}\right]=\min\left[\sqrt{\frac{\sum_{k=1}^{N}{\left|Q_{k}-R_{k}\right|}^{2}}{N}}\right]$$ (3)

where *N* is the number of atoms in a chain, and *δ_k_* is the distance between atom k in the original chain and atom k in the superposed chain. The explicit distance calculation is presented in the right side expression, where {**Q**_*k*_} and {**R**_*k*_} are the sets of coordinates of the original chain and the superimposed chain, respectively.

### Cutting proteins into subunits

Our in-house-designed Perl program, pdbslicer [30], was used to extract the complete subunit and the backbone subunit of 258,822 (= 129,411 × 2) residues from our main set and calculate their CCMs. The double-dimers set produced two sets of 19,679 residue pairs each, taken from the first and second dimer of each PDB file (78,716 residues in total).

## Results and discussion

### Deviation from rotational symmetry

We begin with an overview of the degree of the protein homodimers deviation from *C*_2_ symmetry. To this end, we calculated *S*(*C*_2_) for our main set of 565 homodimers. Descriptive statistics are presented in Table 1 for both the complete set of atoms and the backbone atoms. Two observations emerge from Table 1. First, the presented CSM values are generally small in comparison to the full CSM range of [0,100]. Such values have been shown to be typical of proteins with approximate symmetry [27, 35]. Note that the minimum CSM is not 0 but 0.0017 when all of the atoms of the specific protein are taken into account. In other words, among the 565 dimers of our main set, none had a perfect *C*_2_ symmetry. At the backbone level, one homodimer (histidine acid phosphatase from *Francisella tularensis* with X-ray diffraction resolution 1.5 Å, PDB-ID: 3IT3) did reach a CSM of 0.0000, implying perfect *C*_2_ symmetry of the backbone; however, its *S*(*C*_2_) for the complete set of atoms was 0.0041.

**Table 1:** Descriptive statistics of *S*(*C*_2_) for the homodimer sets.

Attribute	Main set: All Atoms (N = 565)	Main set: Backbone atoms (N = 565)	Double-dimers set: All atoms (N = 160)^a^
**Mean**	0.0965	0.0625	0.0945
**Standard deviation**	0.1626	0.1429	0.2100
**SE of mean**	0.0069	0.0060	0.0166
**Minimum**	0.0017	0.0000	0.0043
**Median**	0.0384	0.0130	0.0307
**Maximum**	1.3508	1.2324	1.4325

^a^For the descriptive statistics of each set of 80 homodimers see S1 Table.

**Fig 2:**
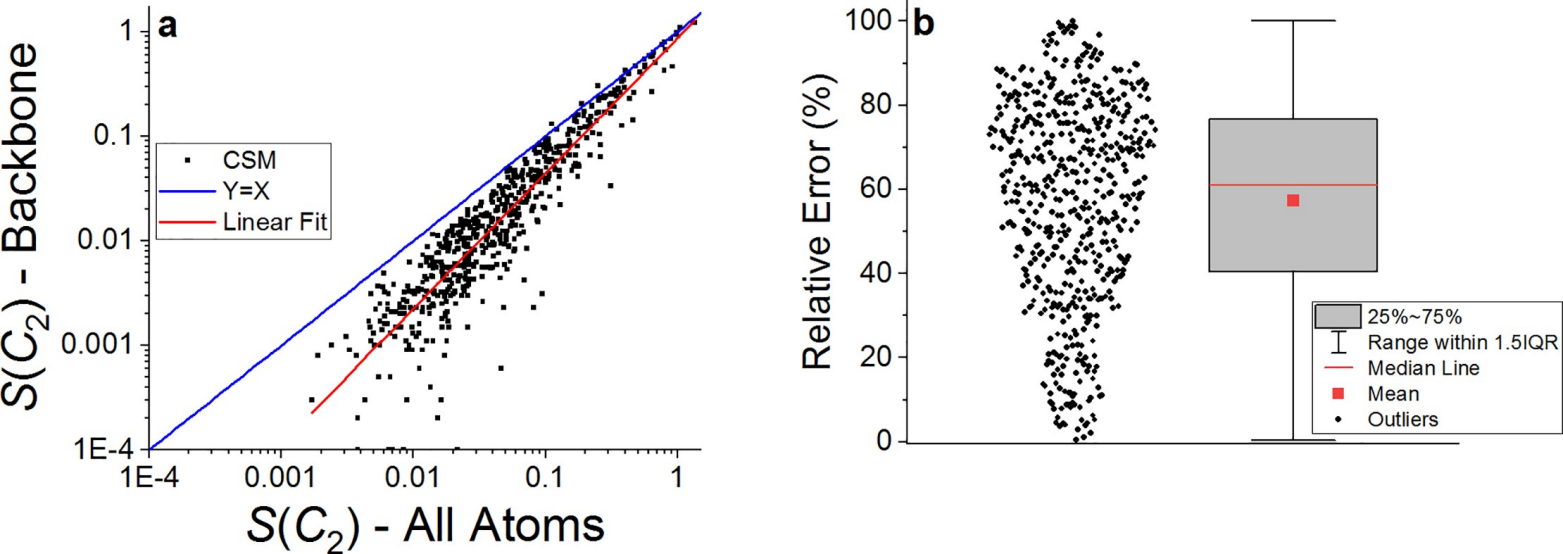
*S*(*C*_2_) for the set of 565 homodimers with approximate symmetry. a. backbone atoms vs. all atoms on a log-log scale. Blue line: the y = x curve. Red line: linear regression curve, log(y) = -0.061 + 1.294∙log(x), R^2^ = 0.85. b. Box and whisker plot of the relative error between *S*(*C*_2_) values calculated for all atoms and the backbone atoms, Relative error = 100∙|*S*(*C*_2_, all atoms)-*S*(*C*_2_, backbone)|/*S*(*C*_2_, all atoms).

The second observation that comes up from Table 1 is that the symmetry of the backbone is generally higher (with smaller CSM values) than that of the complete set of atoms (see Fig 2A). This is to be expected because the backbone of a protein has fewer degrees of freedom than its complete set of atoms due to the well-organized secondary-structure patterns. Although there is a general qualitative correlation between the two measures, the quantitative correlation is not very strong (R^2^ = 0.85, see Fig 2A). Moreover, the relative error between the two measures presented in Fig 2B can be quite high with a median value of 61%.

**Fig 3:**
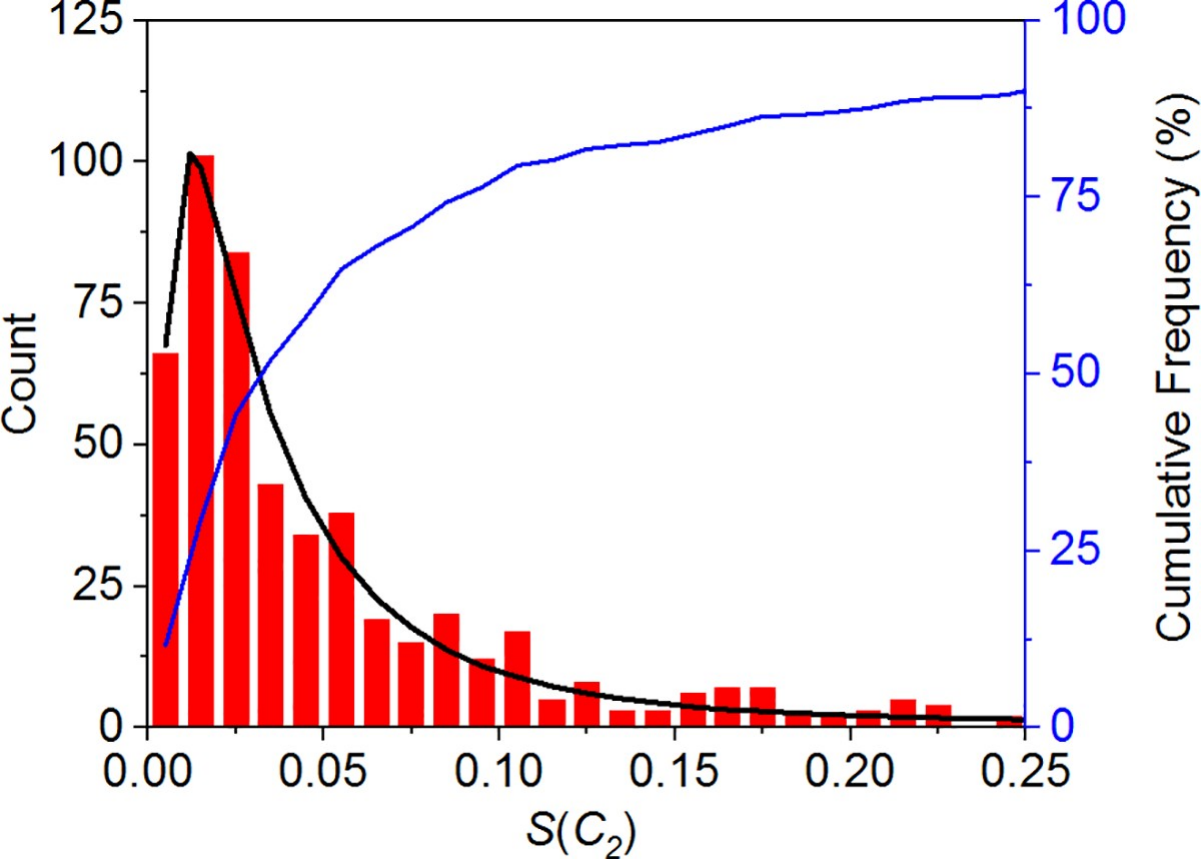
*S*(*C*_2_) distribution for the set of 565 homodimers using all atoms. Bin size was set to 0.01. Blue line–cumulative percent (right scale). Black line–log-normal distribution fitting curve (see fitting details in S1 Table). The right tail of the distribution is hidden to increase visibility. See S1 Fig for the CSM distribution at the backbone level.

Defined as a positive parameter, the CSM distribution is generally characterized by a long tail, as the large standard deviations in Table 1 reveals. These standard deviations indicate that only a few homodimers have relatively large CSM values that shift the distribution and statistical parameters toward a higher CSM. The CSM histogram for the complete set of atoms presented in Fig 3 supports this result (see S1 Fig for the distribution at the backbone level). The blue line (with a scale on the right) presents the cumulative probability of finding a protein with the given distortion level. For 75% of the homodimers, *S*(*C*_2_) < 0.1 (when all atoms are taken into account). At the backbone level, 85% of the homodimers meet this condition. The low CSM values of the vast majority of homodimers imply that in general, *C*_2_ symmetry is approximately preserved.

The black line in Fig 3 represents the fitting of a log-normal distribution function to the histogram. This distribution function, which has also been proven relevant for temperature distributions of CSM values [43], is given by

$$y=y_{0}+\frac{A}{\sqrt{2\pi }wx}\cdot \exp\left[-\frac{{\left[\ln\left(x/xc\right)\right]}^{2}}{2w^{2}}\right]$$ (4)

Here, *y*_0_ is the offset, *xc* is the median value, *w* is the log standard deviation and *A* is the area. The expectation value is given by $\bar{S_{G}\left(T\right)}=xc\cdot e^{w^{2}/2}$. A possible interpretation of this fit is that the deviation from symmetry is distributed in accordance with the principle of maximum entropy [44], namely, that from all the models satisfying the constraints of the data, one should choose the model with the least amount of information [45]. Our results support the notion that the probability distribution which best represents the current state of knowledge on protein symmetry within our dataset is the one with the largest entropy. Given that entropy and symmetry are competing factors in determining protein structure, these results suggest that entropy is the factor preventing proteins from reaching perfect symmetry.

To confirm the validity of the analysis, we calculated *S*(*C*_2_) using all the atoms of the double-dimers set. The analysis was based on 80 proteins for which the biological assembly is a homodimer, but the asymmetric units contained two homodimers. The right hand column of Table 1 provides descriptive statistics of the combined set of 160 homodimers. The level of *C*_2_ symmetry is evidently similar to the main set. A separate examination of each set of 80 homodimers shows a certain variability of *S*(*C*_2_) between copies of the same dimer in a given crystal, which could be attributed to the crystallization process and to small differences in the packing and the environment of each dimer [46] (see S2 Table). However, the distribution of *S*(*C*_2_) in each set is similar (see S2 Fig).

### RMSD and CSM

The RMSD is commonly used to distinguish between symmetric and asymmetric proteins. Several studies have defined RMSD threshold levels above which the proteins are considered asymmetric: Abraham et al. [21] regard protein homomers with RMSD ≤ 0.5 Å as symmetric, while Pednekar and Durani [22] regard proteins with RMSD < 0.2 Å as "more or less perfectly symmetric". Swapna et al. [23] use a threshold similar to that of Abraham et al. [21], but their RMSD is calculated based on α-carbons only. In general, RMSD vaguely correlates to the CSM. In S3 Fig, the RMSD for our main set of protein homodimers is plotted against *S*(*C*_2_). While the RMSD increases with the CSM, there is no direct correlation between the two. This lack of correlation becomes more prominent when the CSM increases, as was shown by Tuvi-Arad and Alon [35]. It results from the different definitions of the two measures, and supports Bonjack-Shterengartz and Avnir's claim [27] that the RMSD does not evaluate the symmetry itself.

### Conformational similarity of equivalent residue pairs

**Fig 4:**
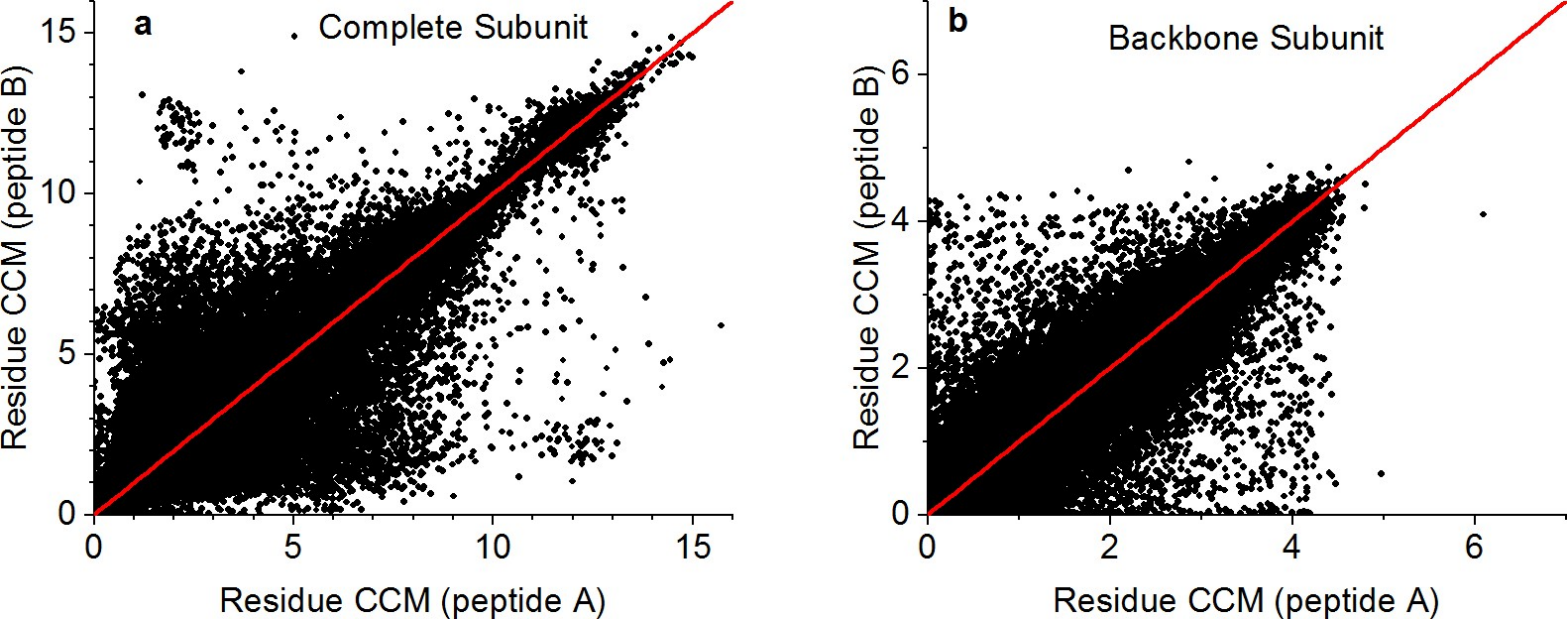
CCM of matching residues for the set of 565 proteins. **a.** Complete subunit. **b.** Backbone subunit. The red line represents the identity line (Y = X).

Given a perfectly symmetric homodimer, we expect the conformation of each residue on one peptide to be equivalent to that of the matching residue on the other peptide (with the same chemical identity and sequence number). The CCM, being a global parameter of the coordinates of a given structure, was used here as a descriptor of the conformational state of each residue with the subunits defined in Fig 1. Fig 4 presents the CCM of 129,411 matching residue pairs in our main set using the complete subunit (Fig 4A) and the backbone subunit (Fig 4B). The red line in each plot represents the identity line (Y = X) for which the CCM difference between matching residues, *d*_i_, is zero, defined as:

$$d_{i}=\left|CCM\left(residue_{i}\left(peptide_{A}\right)\right)-CCM\left(residue_{i}\left(peptide_{B}\right)\right)\right|$$ (5)

Here *i* represents the residue sequence index, and *A* and *B* are peptides of the same protein. Obviously, many pairs of amino acids have very different chirality levels that are attributed to different conformational states, both with and without the side chain atoms. Comparing Fig 4A and 4B, one notes that in both cases there is a similar spread around the identity line, indicating that the different conformation exists already at the backbone level and is not due exclusively to the side chains. Nevertheless, the chirality levels of the backbone atoms are smaller than those of the complete set of atoms. The reason for this may be that less atoms are included in the calculation, and the conformations are confined to the secondary-structure segments of the proteins.

Residue conformations are commonly described in terms of Ramachandran plots and dihedral angles, and are divided into four subgroups: Glycine, Proline, Pre-Proline (i.e., any residue except Gly and Pro preceding Pro) and General (all other residues) [47, 48]. We have redrawn Fig 4 for each of these groups (see S4 and S5 Figs). However, other than the sample size (the number of general residues is much larger than that of Gly, Pro or Pre-Pro), no significant differences emerged in the spread of the CCM values that could be attributed to the type of the Ramachandran group. Notably, despite its description as the only achiral amino acid, Gly is chiral within the protein, with a CCM range of [0,5], consistent with previous studies [30]. Looking again at Table 1 and Fig 3, we can now claim that the *S*(*C*_2_) values of the homodimers should be regarded as a common CSM scale for proteins with approximate symmetry, although they represent only one aspect of the deviation from perfect symmetry. At the residue level, the deviation can be much larger, as is evident from Fig 4.

### Statistical analysis of CCM differences

**Fig 5:**
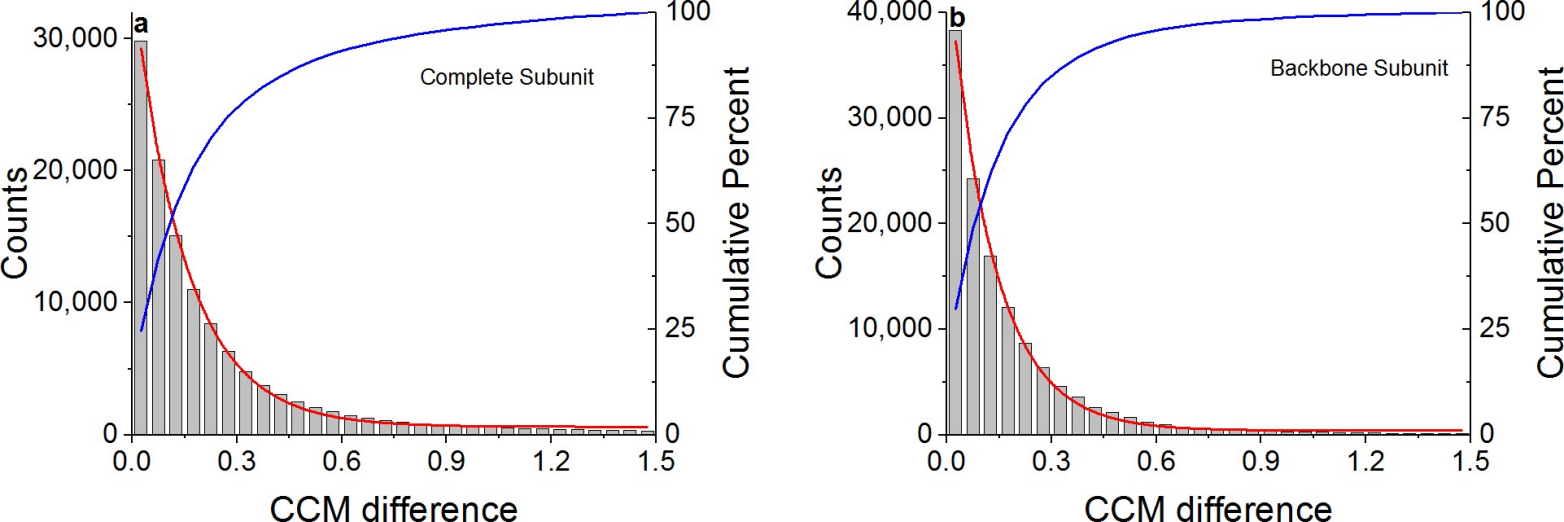
Distribution of CCM differences. a. Complete subunit. b. Backbone subunit. Blue line–cumulative percent (right scale). Red line–exponential distribution fitting (left scale). Bin size was set at 0.05. See fitting details in S3 Table.

**Fig 6:**
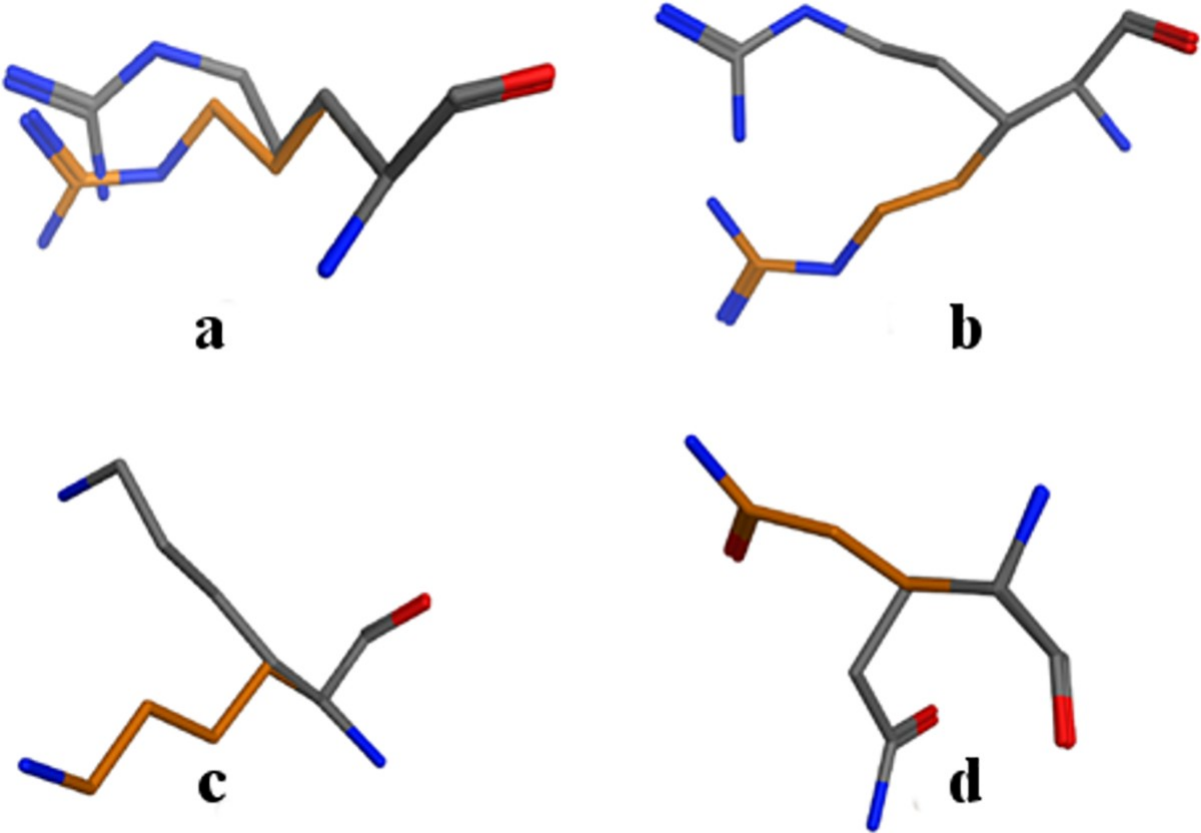
Pairs of residues with various CCM differences (*d*_i_) superposed on each other. The side chain of Chain A is colored orange and the side chain of Chain B is colored gray. Superposition is based on the backbone alignment algorithm implemented by MOE [42]. a. Arg-282 of 4LLS, *d*_i_ = 0.5000. b. Arg-188 of 3X3Y, *d*_i_ = 1.0001. c. Lys-45 of 2Q20, *d*_i_ = 2.5026. d. Gln-32 of 2ECS, *d*_i_ = 5.0027.

As shown in Fig 4, the conformations of residue pairs in our main set may well be significantly different. Histograms of these differences are presented in Fig 5A and 5B for the complete subunit and for the backbone subunit respectively. Table 2 presents the descriptive statistics of the data and Fig 6 gives examples of the structural differences between matching pairs of residues. The main observation that emerges from the data is that in 90% of the complete subunit pairs and 98% of the backbone subunit ones, *d*_i_ < 1. The maximum difference is, however, much larger, ca. 12 for the complete subunit and 4 for the backbone subunit. These results suggest that a relatively small number of residues are responsible for the protein distortion. The chirality level of the third quartile supports this finding: the CCM difference for 75% of the residue pairs is less than 0.36 for the complete subunit and 0.23 for the backbone subunit. At the same time, in 23% of the pairs with the complete subunit and 30% of the pairs with the backbone subunit, *d*_i_ < 0.05, that is, their conformations are relatively similar, albeit not exactly the same. Notably, out of 129,411 residue pairs, the value of 0.000 was obtained for *d*_i_ only 38 times (0.023%) in the complete subunit and 88 times (0.068%) in the backbone subunit. These numbers highlight the extreme rarity of perfect symmetry.

**Table 2:** Descriptive statistics of the CCM difference (N = 129,411).

Attribute	Complete subunit	Backbone subunit
**Mean**	0.4130	0.1918
**Standard deviation**	0.8375	0.2914
**SE of mean**	0.0023	0.0008
**Minimum**	0.0000	0.0000
**1**^**st**^ **Quartile**	0.0552	0.0404
**Median**	0.1460	0.1055
**3**^**rd**^ **Quartile**	0.3601	0.2294
**Maximum**	11.8659	4.4199

Comparing the results obtained for the complete subunit with those of the backbone subunit show that the deviation from symmetry of the complete subunit is generally higher, suggesting that in cases of near symmetry, the side chains play a major role in protein distortion. The CCM difference histograms can be fitted by an exponential decay distribution function related to the Boltzmann distribution. They can be interpreted as the number of possibilities for distributing M pairs of matching residues in N levels of conformational differences. Assuming that a symmetric arrangement is energetically favorable, we expect to find the maximum of such a distribution when the conformational difference is negligible (i.e., a CCM difference approaching zero). The probability of finding pairs with a larger difference is expected to decrease with the increase in conformational difference. From an entropy perspective, however, high conformational differences are the favored state. The final distribution is therefore determined by an interplay between energy and entropy. These results support the findings of Butterfoss and Hermans [49] and of Zhu et al. [50], who suggested that the conformational energy of residues matches a Boltzmann distribution.

### Analysis of distorting residues

**Fig 7:**
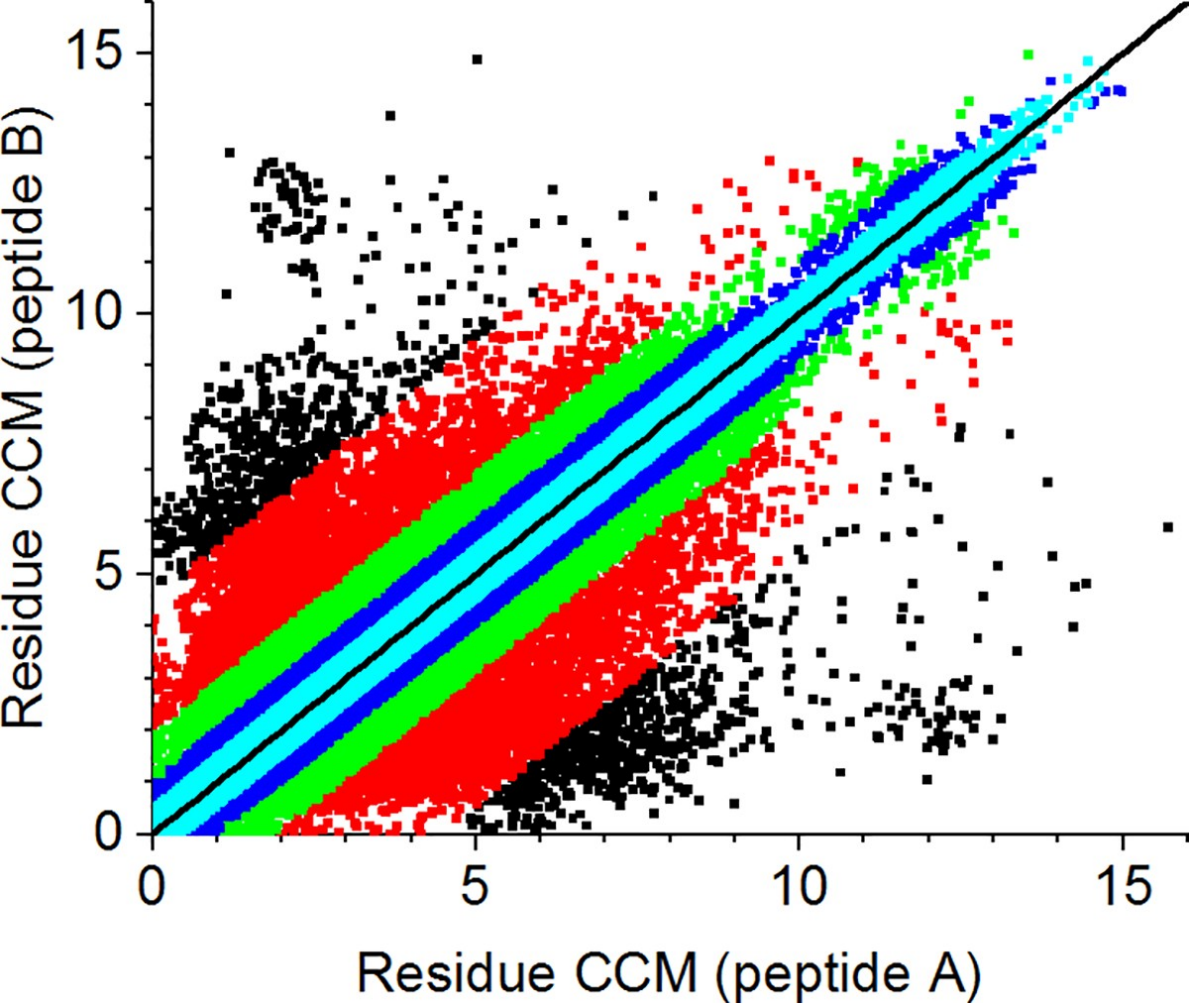
CCM of matching residues for the set of 565 proteins in the complete subunit. Black: the top 1% of distorted pairs (*d*_i_ > 4.48); red: the top 5% of distorted pairs excluding the 1% group (1.87 ≤ *d*_i_ ≤ 4.48); green: the top 10% excluding the 5% group (0.96 ≤ *d*_i_ ≤ 1.87); blue: the top 20% excluding the 10% group (0.46 ≤ *d*_i_ ≤ 0.96); cyan: the rest, 80% of the pairs (*d*_i_ < 0.46). The black line represents the identity line (Y = X).

To determine the residues that cause the highest distortion, we redrew Fig 5 and colored the points according to their CCM difference values. The new plot, presented in Fig 7, uses black dots to represent the top 1% of distorted pairs, red dots for the next 5% of distorted pairs, green dots for the next 10%, blue for the next 20%, and cyan blue for the other 80% of the pairs. One can clearly see that as *d*_i_ decreases, more points are spread over a narrower area, confirming our previous statement that a relatively small number of residues are responsible for the protein distortion.

Following is a statistical analysis of the subset of residue pairs with the highest distortion levels–the 5% of pairs with *d*_i_ > 1.87, that comprises 6,471 residue pairs. Note that here, in contrast to Fig 7, the 5% subset includes the residue pairs of the top 1% subset (with 1,294 residue pairs), which is itself too small for statistical analysis. Table 3 presents the abundance (in counts and percentages) of each residue in the subset and in the general population, and the ratio between them, termed abundance ratio. This ratio was used as a sorting parameter in Table 3. The data is divided into two groups of amino acids: group I is comprised of residues for which the abundance ratio is higher than 1, and includes 9 amino acids listed in a descending order of ratios: Lys, Gln, Glu, Arg, Asn, Met, Ser, Thr and Asp; group II is comprised of the remaining 11 amino acids, for which the abundance ratio is lower than 1: Ile, Pro, His, Val, Cys, Leu, Trp, Tyr, Phe, Ala and Gly.

**Table 3:** Abundance of amino acid pairs in the subset of the 5% most distorted pairs and the general population. Counts represent the number of matching residue pairs. Percentages are relative to the subset.

	Amino acid	Top 5% (*d*_i_ >1.87) (N = 6,471)		General population (N = 129,411)		Abundance ratio
		Count	%	Count	%	
**Group I**	**Lys**	1,069	16.52	6,638	5.13	3.22
	**Gln**	623	9.63	4,675	3.61	2.67
	**Glu**	942	14.56	8,181	6.32	2.30
	**Arg**	598	9.24	6,395	4.94	1.87
	**Asn**	444	6.86	5,248	4.06	1.69
	**Met**	204	3.15	2,477	1.91	1.65
	**Ser**	588	9.09	7,260	5.61	1.62
	**Thr**	393	6.07	6,970	5.39	1.13
	**Asp**	428	6.61	7,704	5.95	1.11
**Group II**	**Ile**	267	4.13	7,423	5.74	0.72
	**Pro**	207	3.20	6,142	4.75	0.67
	**His**	70	1.08	3,214	2.48	0.44
	**Val**	196	3.03	9,481	7.33	0.41
	**Cys**	24	0.37	1,496	1.16	0.32
	**Leu**	154	2.38	11,766	9.09	0.26
	**Trp**	17	0.26	1,893	1.46	0.18
	**Tyr**	39	0.60	4,388	3.39	0.18
	**Phe**	46	0.71	5,386	4.16	0.17
	**Ala**	104	1.61	12,253	9.47	0.17
	**Gly**	58	0.90	10,421	8.05	0.11

Differently from the complete set of atoms, the abundance of residues in the top 5% group at the backbone level is quite similar to their abundance in the general population, with an abundance ratio in the range of 0.9–1.25. This result supports the assumption that the side chains cause most of the distortion. Nevertheless, significant conformational differences at the backbone level may result in a mismatch of the secondary-structure annotation of the two chains, as determined by the DSSP method [40]. In the main set, we found different secondary-structure annotations for 3% of the residue pairs on average. Nevertheless, the most common secondary structures, α-helix and β-sheets, which are relatively rigid compared to the other secondary structures, had a lower mismatch percentage (~1%), whereas for the less common secondary structures, the mismatch reached up to 7%. S4 Table specifies the details.

**Fig 8:**
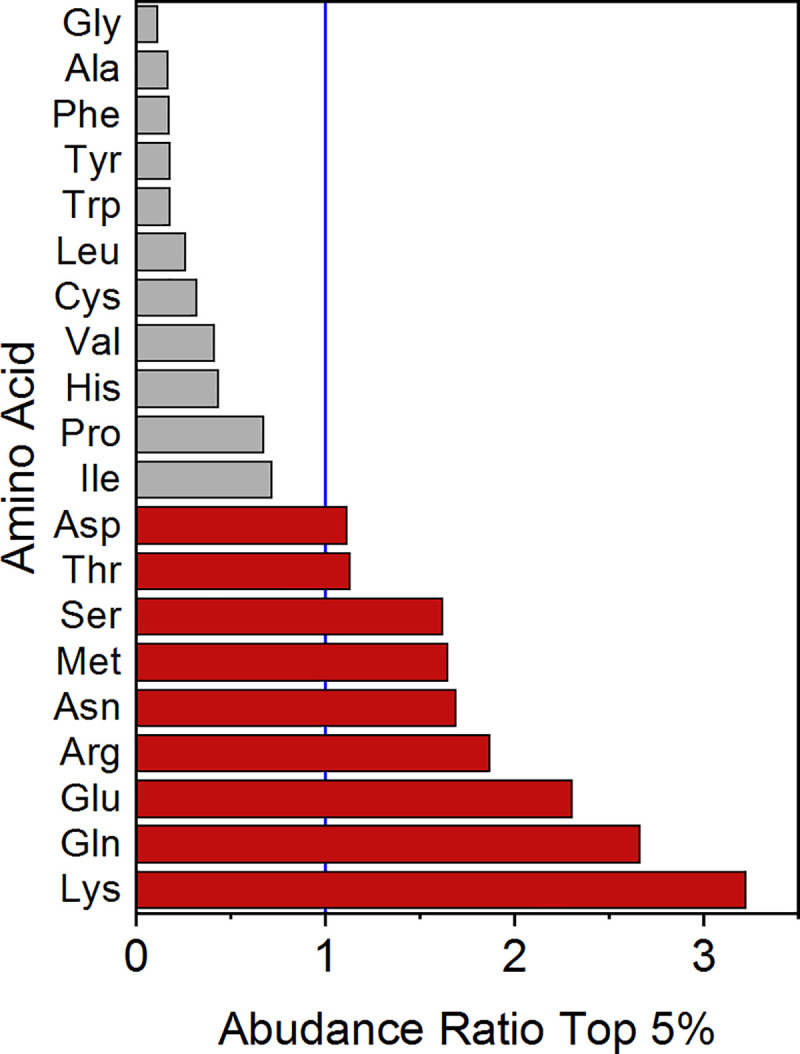
Abundance ratio of amino acids in the subset of the top 5% most distorted residue pairs relative to the general population. Blue line marks an abundance ratio of 1. Red bars: amino acids with abundance ratio >1. Gray bars: amino acids with abundance ratio <1.

Fig 8 is a bar chart presentation of the abundance ratio number of each amino acid listed in Table 3. The blue vertical line represents a ratio of 1, the red bars represent amino acids of the first group (with an abundance ratio >1) and the gray bars represent amino acids of the second group (abundance ratio <1). Notably, all the pairs in the top 5% subset contribute to high protein distortion. However, the amino acids in group I occur in relatively high percentages compared to their abundance in the general population, leading to the conclusion that the contribution of these amino acids to the distortion is significantly higher. One might ask what the common characteristic of the amino acids in group I is to make them cause the distortion. We elaborate on this in the next section.

### Common characteristics of distorting residues

As Fig 8 shows, Group I, with a high tendency for distortion, includes Lys, Gln, Glu, Arg, Asn, Met, Ser, Thr, and Asp (see S6A Fig). Group II, with a low tendency for distortion, includes Ile, Pro, His, Val, Cys, Leu, Tyr, Trp, Phe, Ala, and Gly (S6B Fig). The two groups differ in their structure: Group I consists of amino acids with long, charged, or polar side chains that are all commonly found on the surface of the protein. Group II typically consists of the hydrophobic amino acids commonly buried inside the protein core (Ile, Pro, Val, Leu, Tyr, Phe, Ala and Gly) [51–53]. Several polar amino acids (His, Cys, Trp) were found in group II but not in group I, while Met, a non-polar hydrophobic amino acid, was found in group I but not in group II.

The parameters that influence the tendency of amino acids to distort the protein deserve further exploration. Residues with long side chains, such as Lys, Gln, Glu and Arg, have more degrees of freedom to rotate in space than those with short side chains. The probability that two equivalent residues with long side chains will crystalize in exactly the same conformation on the two peptides of a homodimer is thus low. It is therefore not surprising to find such amino acids in group I. That being said, we would not expect residues with short side chains (e.g., Gly and Ala) to necessarily have the same conformation on both peptides. However, the discrepancies in their conformations, as quantified by the CCM difference, are far smaller, justifying their classification as belonging in group II.

The polarity of the side chains is another distorting factor, as it allows residues to create rather strong nonbonding interactions with the solvent or with ligand molecules, possibly contributing to a conformational change that breaks the symmetry. In other words, the external environment of a given residue is an important factor dictating the conformation of its side chain, and may cause an overall symmetry distortion if it is different for two equivalent residues on both peptides. However, the presence of a polar residue is not sufficient as the length of the side chain and its polarity both influence the distortion tendency. Comparing Cys and Met, the first is more polar while the second has a longer side chain. Cys tends to create disulfide bonds that stabilize the protein structure and confine its geometrical conformation. On the other hand, the classification of Met in terms of polarity can be ambiguous: the literature describes it as either nonpolar, polar, or weakly polar [54]. Nevertheless, its length increases its ability to rotate in space, making it a good candidate for breaking the symmetry of the protein. The study of Yuan et al. [55] further supports this point. They calculated the number of minima for each amino acid in a model molecule of the form CH_3_C(O)-Res-N(H)CH_3_, where Res is replaced with various amino acids. They showed that the number of Met minima is comparable to that of Arg (57 and 61 respectively, at the B3LYP/apc-1 level), and both are significantly higher than those of all the other amino acids (the third residue in the list is Lys with 39 minima). It is worth noting that the number of minima of the model molecule is not necessarily the same as in a real protein sequence (e.g., internal hydrogen bonds are more probable in the model molecule). Nevertheless, the differences found by Yuan et al. [55] for Met are significant. Similarly, despite their polarity, Trp and His belong with group II since from a structural perspective the aromatic rings in their side chains have a steric effect that decreases the number of structural conformations. Accordingly, Yuan et al. [55] found the number of minima of Trp and His to be smaller than that of Phe, which has a relatively similar structure (Trp has 26 minima in the model molecule, His has 24 and Phe has 30, at the B3LYP/apc-1 level).

The division into groups I and II is by no means dichotomous. We expect residues with abundance ratios close to 1.0 to transfer to the other group when the sample size changes. Focusing on the 10% residue subgroup with the highest CCM difference (see S5 Table), Pro moves from group II to group I, while Ser and Thr move from group I to group II. Pro is generally described by a confined geometry (as expressed in its Ramachandran plot) [47]. However, it is also polar and as explained above, the interplay between the side chain's rotational flexibility in space and its polarity dictates its classification. The classification of Ser and Thr can be similarly justified. Note, however, that the classification of all other amino acids remains unaffected by their transfer from the 5% group to the 10% group, validating our original classification.

An interesting question to investigate regards the effect of neighboring amino acids on the conformation of a single residue. One way to explore this effect is to examine the location of the distorting residues along the relevant secondary structure segment. In a previous study [31] we found that residues with outlier CCM values are likely to be found at junctions between two secondary structure segments, where the folding pattern forces them to distort. As a crude approximation, we defined a residue location as "edge" if it was the first or last residue of a secondary structure segment, and "middle" otherwise. The secondary structure designation was based on the first peptide of each protein (ignoring the mentioned discrepancies between the two peptides). Analysis of our main dataset showed that 51% of all the residues, and 54% of the residues in the 5% most distorting residues could be designated "edge". A more detailed analysis of the 5% most distorting residues revealed more "edge" residues in random coils, bends and turns segments (78%, 87% and 90% respectively) as against α-helices (21%) and β-strands (44%). This could be attributed to the fact that coils, bends, and turns segments are generally shorter than helices and strands segments. Compared to the general population, these numbers deviate by only ±2–3%, a difference that seems too small to be considered significant. In other words, the 5% most distorting residues are equally likely to be found at the edge or in the middle of a secondary structure segment, and the neighboring amino acids cannot explain their high distortion levels.

**Fig 9:**
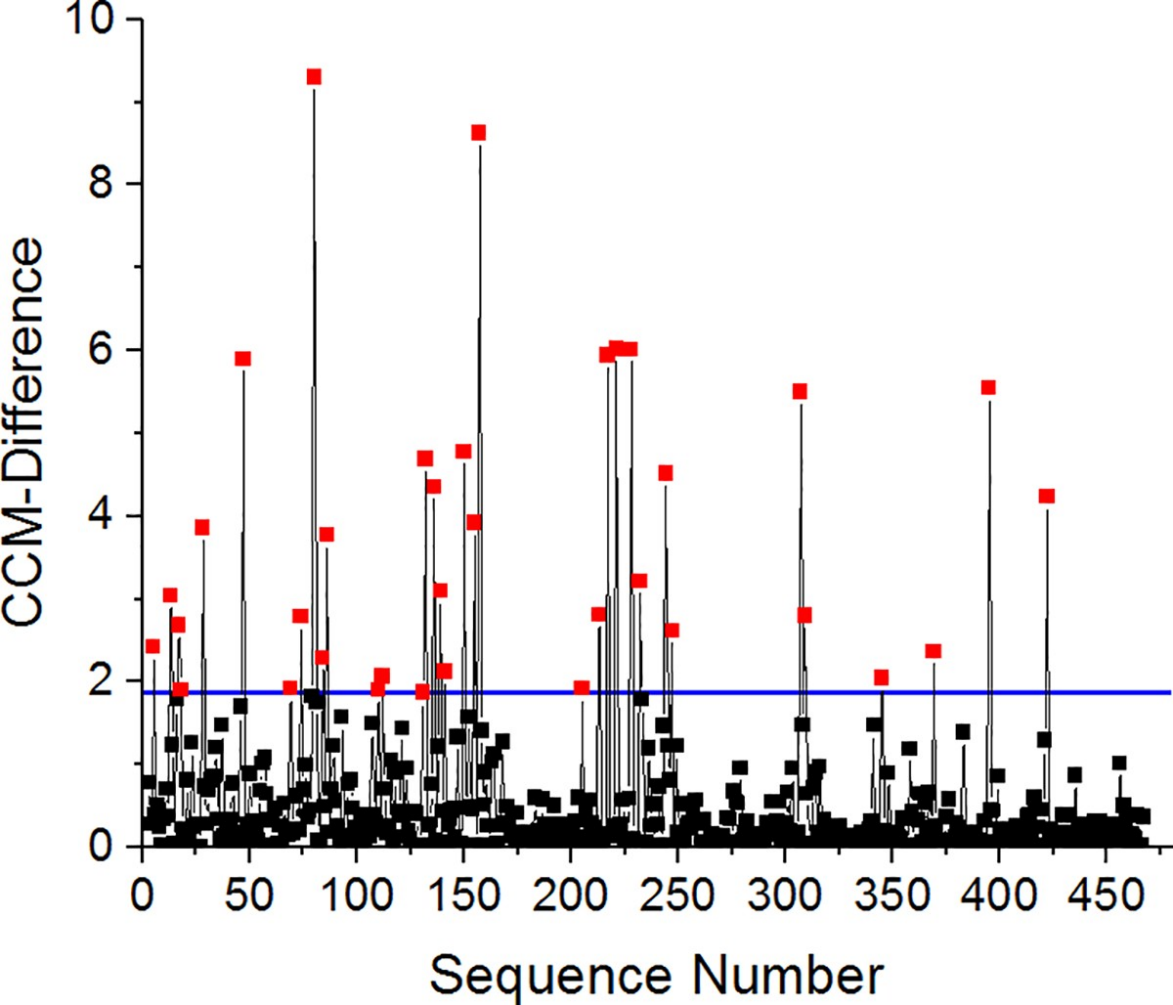
CCM difference spectrum for the protein 6-Phosphogluconate dehydrogenase (PDB ID: 3FWN) taken from the main set. Horizontal blue line represents the *d*_i_ limit of the 5% most distorting residues. Red: residues with high distortion level. Black: residues with low distortion level.

Another way to test the influence of neighboring amino acids is to plot a spectrum of the CCM difference along the sequence of a single protein. Fig 9 displays such a plot for the protein 6-Phosphogluconate dehydrogenase (PDB ID: 3FWN). In this spectrum, sharp red peaks represent pairs with high discrepancies in CCM level. As is evident from the plot, these peaks are spread throughout the protein sequence and are relatively isolated, while their neighboring residues show much smaller CCM differences. Similar plots drawn for other proteins in our dataset support the notion that while neighboring residues play an important role in constructing a symmetric homodimer in the first place, they are unable to explain the anomalous differences of side chains conformations which are the focus of this study. Moreover, as suggested by Swapna et al. [23], highly distorted residues are spread over the entire protein. All the same, a detailed analysis that takes into account non-bonding interactions and solvent effects would possibly be able to provide a fuller explanation of anomalous local distortions.

**Fig 10:**
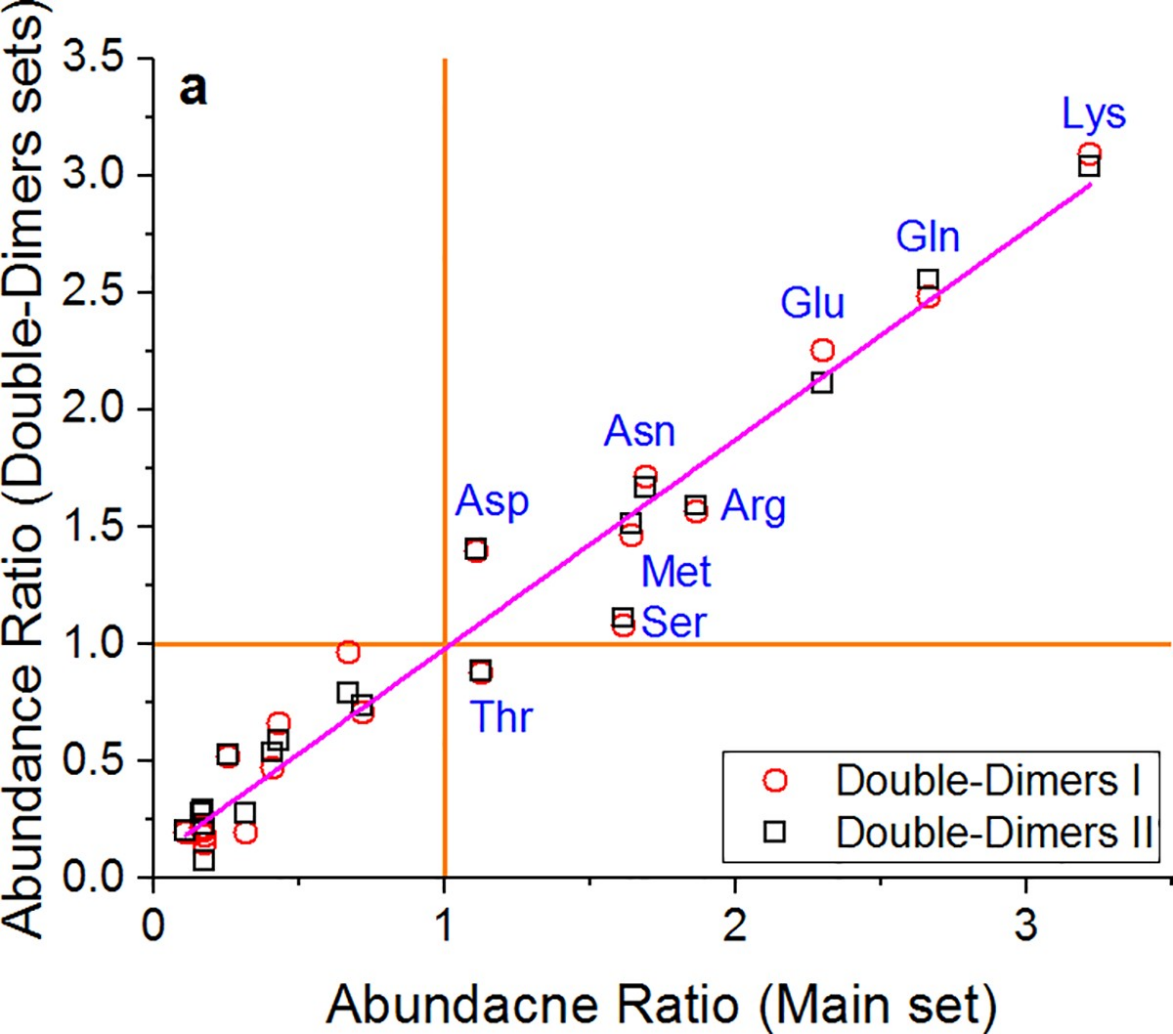
Abundance ratio of the two double-dimers sets versus the main set. Orange lines represent an abundance ratio of 1. **a.** The. Red circles: set I, black squares: set II. Magenta line: linear fitting (Y = 0.08+0.89X, R^2^ = 0.95). Distorting residues are marked in blue based on the main set.

Finally, below is the analysis of the two double-dimer sets used as control data sets and defined in the Methods section. The data of the 10% most distorting residues of these sets were analyzed in a way that complied with two requirements: a large enough sample for statistical analysis, and a reasonable *d*_*i*_ threshold defining the most distorting residues. The results confirm that the classification generally holds for the control set, with small differences in the order of amino acids in each group. Fig 10 displays a plot of the abundance ratio of the double-dimer control set (top 10% subset) vs. the abundance ratio of the main set (top 5%). The plot reveals that while some differences exist between the two double-dimer sets, they are relatively small, and do not influence the general distortion trend. In other words, the effects of crystal formation and packing on the protein's symmetry are minor compared with the general conformational flexibility of specific residues. Similar to the main set, in these sets too Lys, Gln, Glu, Asn, Arg, Met, Asp and Ser belong in group I with an abundance ratio larger than 1. Thr transfers to group II with an average abundance ratio of 0.88 which is close to 1. Interestingly, the maximum CCM difference obtained throughout our data is that of Thr residues, reaching a value of 11.9 –higher than the maximum distortion of all others residues. Moreover, the abundance ratio of Thr in the general population is between 5–6% in all the datasets, but in the top 1% group its abundance is much higher and stands at 13–15%. This suggests that Thr is highly flexible, and can create a significant local asymmetry, justifying its classification in group I. Details of the abundance ratios in the double-dimer sets are provided in S6 and S7 Tables.

## Conclusions

The methodology of continuous symmetry and chirality measures offers accurate and efficient tools to describe protein structure, both for the whole protein and at the residue level. This method of quantification helps understand where and why proteins fail to reach perfect symmetry, and what amino acids are responsible for this failure. This study analyzed the symmetry of 565 homodimers taken from very accurate X-ray measurements. The analysis highlighted the contribution of the side chains to the overall symmetry of the protein, and showed that a relatively small portion of the residues (up to 10%) causes the deviation from perfect symmetry. Our findings suggest that amino acids form two groups with different tendency to distort the symmetry of protein homomers in cases of near symmetry. Group I residues (Lys, Gln, Glu, Arg, Asn, Met, Ser, Thr and Asp) have long or polar side chains that are likely to disrupt the symmetry of the protein homodimer. Group II residues (Ile, Pro, His, Val, Cys, Leu, Trp, Tyr, Phe, Ala and Gly) have a short or aromatic side chain that enhances the protein's symmetry. The division into groups I and II is not very strict, and as demonstrated above, we expect the classification of amino acids with marginal properties to change when the sample size changes. Nevertheless, these results imply that there is a direct link between the structural flexibility of the side chain and the breaking of protein homodimers symmetry. We have also showed that the highly distorted residues stand quite far apart from their neighbors, and are spread throughout the sequence. These findings give rise to the conclusion that in cases of near symmetry, entropic considerations control the final confirmation of proteins in a crystal. The present study substantiates previous studies of much smaller datasets, e.g., Swapna et al. [23], who claimed that the asymmetry is spread over the entire protein, and Pednekar and Durani [22], who attributed the breaking of symmetry to polar or aliphatic side chains. Given the large size of our dataset, it is reasonable to expect that similar results would be obtained for protein homomers with higher rotational symmetry.

The proposed characterization of amino acids is a step forward in our attempt to understand the role played by each residue in determining the structure and function of protein homomers. The tools presented here can pave the way for further exploration of the sources of distortion, for example with regard to regions of local distortion, the effect of ligands, solvent, experimental conditions and methodologies, and the overall function of proteins. Furthermore, this type of analysis can be used to characterize the three-dimensional structure of proteins in solid-state or in a solution, to analyze conformational changes during dynamical processes, and to explore symmetry related quantitative structure–activity relationships.

## Supporting information

S1 Appendix List of PDB-IDs used in this study. (DOCX)

S1 Fig *S*(*C*_2_) distribution of the set of 565 homodimers using the backbone atoms. Bin size was set to 0.001. Blue line–cumulative percent (right scale). Black line–log-normal distribution fitting curve (see fitting details in S1 Table). The right tail of the distribution is hidden to increase visibility.
(TIF)

S2 Fig *S*(*C*_2_) distribution of the two sets of 80 homodimers of the double-dimers set. Bin size was set to 0.05. The right tail of the distribution is hidden to increase visibility.
(TIF)

S3 Fig RMSD calculated for all atoms vs. *S*(*C*_2_). **a.** ca. 90% of the homodimers in our data set. **b.** All proteins in the main set.
(TIF)

S4 Fig CCM of matching residue pairs, divided according to the Ramachandran subgroups calculated with the complete subunit. **a.** General, **b.** Gly, **c.** Pro, **d.** Pre-Pro. Red line: Y = X curve.
(TIF)

S5 Fig CCM of matching residue pairs, divided according to the Ramachandran subgroups calculated with the backbone subunit. **a.** General, **b.** Gly, **c.** Pro, **d.** Pre-Pro. Red line: Y = X curve.
(TIF)

S6 Fig Amino acids ordered by their distortion tendency. **a.** Amino acids with high tendency to distort the symmetry. **b.** Amino acids with low tendency to distort the symmetry. Side-chains are marked in red for clarity.
(TIF)

S1 Table Fitting parameters of the log-normal distributions in Fig 3 and S1 Fig. (DOCX)

S2 Table Descriptive statistics of *S*(*C*_2_) for the double-dimers set. (DOCX)

S3 Table Fitting parameters for the exponential decay distributions of Fig 5. (DOCX)

S4 Table Mismatch of secondary-structure annotation. (DOCX)

S5 Table Amino acid abundance in the subset of the 10% most distorted pairs, of the main set, compared to the general population. Counts represent number of matching residue pairs. Percentages are relative to the subset.
(DOCX)

S6 Table Amino acid abundance in the subset of the 10% most distorted pairs compared to the general population of the first subset of double-dimers. Counts represent number of matching residue pairs. Percentages are relative to the subset.
(DOCX)

S7 Table Amino acid abundance in the subset of the 10% most distorted pairs compared to the general population of the second subset of double-dimers. Counts represent number of matching residue pairs. Percentages are relative to the subset.
(DOCX)
