# Supplementary material for: Side chain flexibility and the symmetry of protein homodimers
Source: PLoS One. 2020 Jul 24;15(7):e0235863. doi: 10.1371/journal.pone.0235863 (PMC7380632; doi:10.1371/journal.pone.0235863)
Supplement: S1 Appendix — (DOCX) [file pone.0235863.s001.docx]

### S1 Appendix: List of PDB-IDs used in this study

**a. Main set: Homodimers with approximate *C*_2_ symmetry (N = 565)**

1C9O, 1CRU, 1E19, 1E7L, 1EAJ, 1EK6, 1ET1, 1EZG, 1FLM, 1GQI, 1GVE, 1GYO, 1GYX, 1H41, 1HW1, 1I6W, 1IX9, 1JUB, 1K20, 1K38, 1KDG, 1KKO, 1KQP, 1KTN, 1L6R, 1LQ9, 1M1F, 1M2D, 1M4I, 1M6J, 1MKK, 1MWQ, 1MXR, 1NEY, 1NKI, 1NWW, 1NXM, 1O1H, 1OC2, 1OF8, 1OFN, 1OFZ, 1OI6, 1OKI, 1PSR, 1PYZ, 1Q6O, 1QLW, 1S0P, 1SBY, 1SH8, 1SR7, 1T7H, 1TU7, 1TZP, 1U0K, 1U1W, 1U7I, 1UCR, 1UWK, 1UWW, 1VL7, 1VR7, 1W23, 1W2I, 1WKQ, 1WPN, 1X9H, 1X9I, 1XRK, 1Y37, 1YPQ, 1YUZ, 1YV1, 1Z72, 1Z9P, 1ZMK, 1ZRS, 1ZUY, 2A35, 2AIB, 2AL1, 2AQP, 2AXW, 2C5A, 2C61, 2CAR, 2CXN, 2DCT, 2DF8, 2DSK, 2E5F, 2E6F, 2ECS, 2EJL, 2F22, 2F62, 2FTR, 2FYF, 2G84, 2GLZ, 2GRH, 2GUD, 2GYQ, 2HHJ, 2HIN, 2HZL, 2I3D, 2I51, 2I8T, 2IBD, 2IK9, 2IPR, 2J73, 2J8M, 2J8W, 2J9B, 2JAE, 2JHF, 2NLV, 2NUO, 2OB3, 2OFC, 2OR3, 2ORD, 2P2S, 2P7O, 2PEB, 2PHN, 2PL7, 2PRV, 2Q0L, 2Q20, 2Q8X, 2QE8, 2QIF, 2QL8, 2QZC, 2R1U, 2R5O, 2R8O, 2R8Q, 2RC8, 2UVK, 2V27, 2V6K, 2V9B, 2VD8, 2VHA, 2W1V, 2W2A, 2W31, 2W3P, 2W6A, 2WTP, 2WU9, 2X02, 2X2A, 2XGU, 2XHF, 2XI8, 2XIU, 2XWL, 2Y53, 2YNA, 2Z26, 2Z6R, 2ZDP, 2ZEW, 2ZGW, 3AYJ, 3AZD, 3B0F, 3B12, 3B4U, 3BED, 3BJE, 3BXU, 3C8E, 3C9U, 3CBY, 3CCD, 3COV, 3CP7, 3CTP, 3CWR, 3CZ1, 3CZZ, 3D5P, 3DA8, 3DBP, 3DSB, 3E10, 3E2D, 3E4V, 3E8O, 3EDN, 3EO6, 3EPW, 3ER7, 3F1L, 3FCX, 3FGV, 3FVS, 3FWN, 3G02, 3G16, 3G1P, 3G46, 3G4E, 3GFA, 3GIU, 3GIX, 3GMG, 3GR3, 3GRD, 3GVE, 3GZR, 3H7F, 3HM4, 3HUP, 3HV2, 3I24, 3IJL, 3IT3, 3ITF, 3IX3, 3JUM, 3K67, 3KE7, 3KGY, 3KH1, 3KIZ, 3KUV, 3L46, 3LAS, 3LED, 3LF5, 3LIO, 3LS9, 3LVA, 3LVC, 3LYD, 3LZL, 3MCW, 3MJO, 3MMH, 3MUX, 3MVG, 3MWJ, 3NDO, 3NPK, 3NUA, 3OFG, 3OHE, 3OQ2, 3OQP, 3OXP, 3OZY, 3PMC, 3PPL, 3PWK, 3Q39, 3R41, 3RJT, 3RPE, 3RQ9, 3RUO, 3SJ3, 3SK2, 3SLZ, 3SZA, 3SZJ, 3TA6, 3TAK, 3THQ, 3TKF, 3TN4, 3U2V, 3U6G, 3U7R, 3UB6, 3UEJ, 3UFE, 3UPL, 3URR, 3V9K, 3VCX, 3VE9, 3VK5, 3VM9, 3VRC, 3VSY, 3VV1, 3VW9, 3W5S, 3W6W, 3W86, 3WG3, 3WMT, 3WMV, 3WQC, 3WUR, 3WVA, 3X3Y, 3ZJL, 3ZXC, 4A6R, 4AO9, 4AXO, 4AZJ, 4B4U, 4B8X, 4BF5, 4BRC, 4BZP, 4C5C, 4C72, 4CCQ, 4CHI, 4CI7, 4CNN, 4DN2, 4DO4, 4DQ6, 4DT5, 4DUQ, 4E3X, 4EAE, 4EBG, 4EDH, 4EGU, 4EIV, 4EMT, 4EP4, 4EQS, 4EU9, 4EVU, 4EZG, 4F0B, 4F1W, 4F5L, 4F66, 4FZO, 4G41, 4G6C, 4GCI, 4GEV, 4GNV, 4GUJ, 4H30, 4H3U, 4H7P, 4HHP, 4HMS, 4HWV, 4HZ2, 4I0B, 4I1I, 4I4O, 4I6R, 4IBG, 4ID0, 4IGJ, 4IHZ, 4IIY, 4IJ5, 4INC, 4INE, 4IO2, 4IQB, 4ITB, 4IX3, 4IYJ, 4J42, 4J8C, 4JE1, 4JF1, 4JH2, 4JOS, 4JVU, 4JXR, 4KAE, 4KAL, 4KEM, 4KH7, 4KN8, 4KQK, 4KR5, 4LLS, 4LUC, 4LV6, 4LVF, 4LXQ, 4MAK, 4MAM, 4MAQ, 4MUZ, 4MVA, 4NAX, 4NDS, 4NOG, 4NSV, 4O0C, 4OI3, 4OKI, 4P5E, 4P5N, 4PIC, 4PRS, 4PXE, 4PYQ, 4PZK, 4Q27, 4Q7E, 4Q7O, 4Q9B, 4QIU, 4QTG, 4QUS, 4R3N, 4R8H, 4RDJ, 4RDL, 4RGD, 4RL0, 4RLZ, 4RP3, 4RT5, 4RUQ, 4TLJ, 4TR6, 4TVO, 4U3Y, 4UAB, 4ULV, 4UNU, 4UP3, 4UTU, 4UU3, 4UUL, 4V15, 4WJT, 4WLH, 4WYD, 4X06, 4X1Z, 4X24, 4X7Y, 4XBA, 4XDG, 4XIN, 4XJO, 4XMQ, 4XMR, 4XO6, 4XQ4, 4XQC, 4XYB, 4YAG, 4YEP, 4YKI, 4YNH, 4YPO, 4YX1, 4Z27, 4Z39, 4Z9H, 4ZBD, 4ZFV, 4ZGW, 4ZO2, 4ZR8, 4ZUR, 4ZVC, 4ZWV, 5A9G, 5ACS, 5AIF, 5B7G, 5B89, 5BR4, 5C04, 5C40, 5C5Z, 5CPM, 5CR4, 5DZS, 5E4B, 5EHM, 5EMT, 5EO6, 5EPW, 5F47, 5F5N, 5F6R, 5FI3, 5FQ9, 5FT3, 5G4I, 5GNF, 5GSM, 5GXX, 5GY7, 5HDM, 5HEE, 5HHJ, 5HHT, 5I0X, 5I1U, 5I5M, 5I6A, 5I90, 5IDB, 5IN1, 5IPY, 5J4F, 5J90, 5JAZ, 5JHX, 5JSQ, 5K2I, 5K87, 5KHM, 5KM9, 5KO4, 5KVS, 5L6Q, 5LKB, 5LKV, 5LVS, 5LXE, 5M33, 5M4Z, 5MAU, 5MC1, 5N4K, 5O9M, 5SY4, 5T2Z, 5T55, 5TOQ, 5TQI, 5U0I, 5U4Q, 5U4S, 5UEJ, 5UJP, 5V01, 5V8S, 5V91, 5VN4, 5VSC, 5W8O, 5W8Q, 5WA9, 5WWD, 5X40, 5XAV, 5XFD, 5YN4, 6AMG, 6AQJ, 6B6U, 6ES9.

**b. Double dimer set: Homodimers with *C*_2_ symmetry, 4 chains in the asymmetric unit and 2 chains in the biological assembly (N=80)**

1g2y, 1m7g, 1ouw, 1qh4, 1r0m, 1r2r, 1rmt, 1z9n, 1zkp, 1zmi, 2a2a, 2cjt, 2gcu, 2gz4, 2hox, 2imj, 2odk, 2p8i, 2pwo, 2r6u, 2uvo, 2vba, 2vk8, 2vv6, 2w40, 2xuv, 2yim, 3daq, 3ewz, 3hf5, 3hhp, 3hjb, 3hx8, 3i0y, 3k6m, 3nyu, 3rob, 3rpc, 3sbf, 3siy, 3tg0, 3tnl, 3uxj, 3w0t, 4c5k, 4e0k, 4fbi, 4fgl, 4i4s, 4j7a, 4lrq, 4m0k, 4qrn, 4qyt, 4r81, 4wmj, 4x84, 4y89, 4zba, 5dwm, 5eqa, 5ffx, 5kp1, 5mh2, 5mh6, 5msa, 5nna, 5o95, 5u23, 5u8u, 5v6j, 5w1n, 5w4a, 5zmu, 6am7, 6bhk, 6fkw, 6i3q, 6onc, 6qvf.
