# Supplementary figures and images for: Side chain flexibility and the symmetry of protein homodimers
Source: PLoS One. 2020 Jul 24;15(7):e0235863. doi: 10.1371/journal.pone.0235863 (PMC7380632; doi:10.1371/journal.pone.0235863)

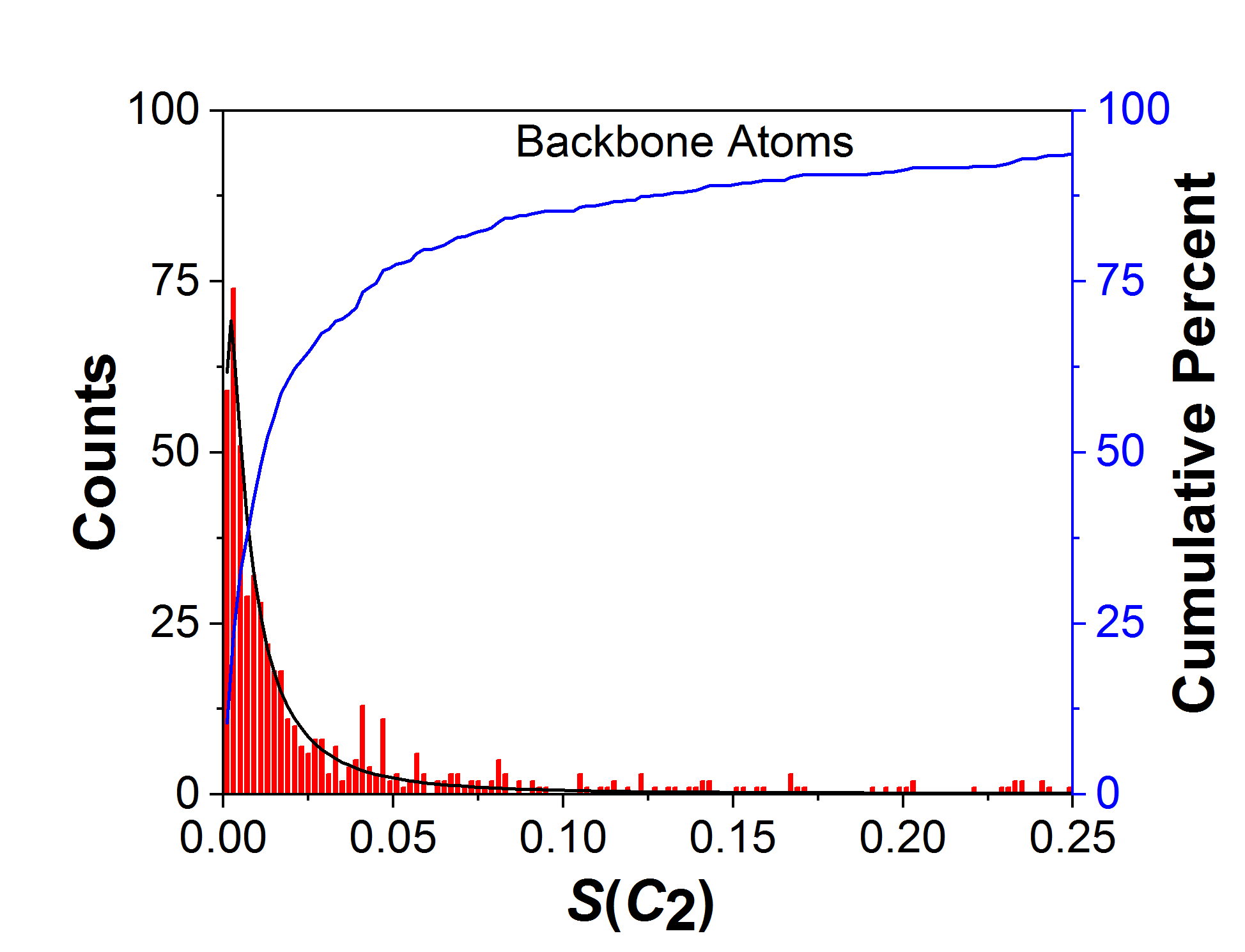

Supplement: S1 Fig — Bin size was set to 0.001. Blue line–cumulative percent (right scale). Black line–log-normal distribution fitting curve (see fitting details in S1 Table). The right tail of the distribution is hidden to increase visibility. (TIF) [file pone.0235863.s002.tif]

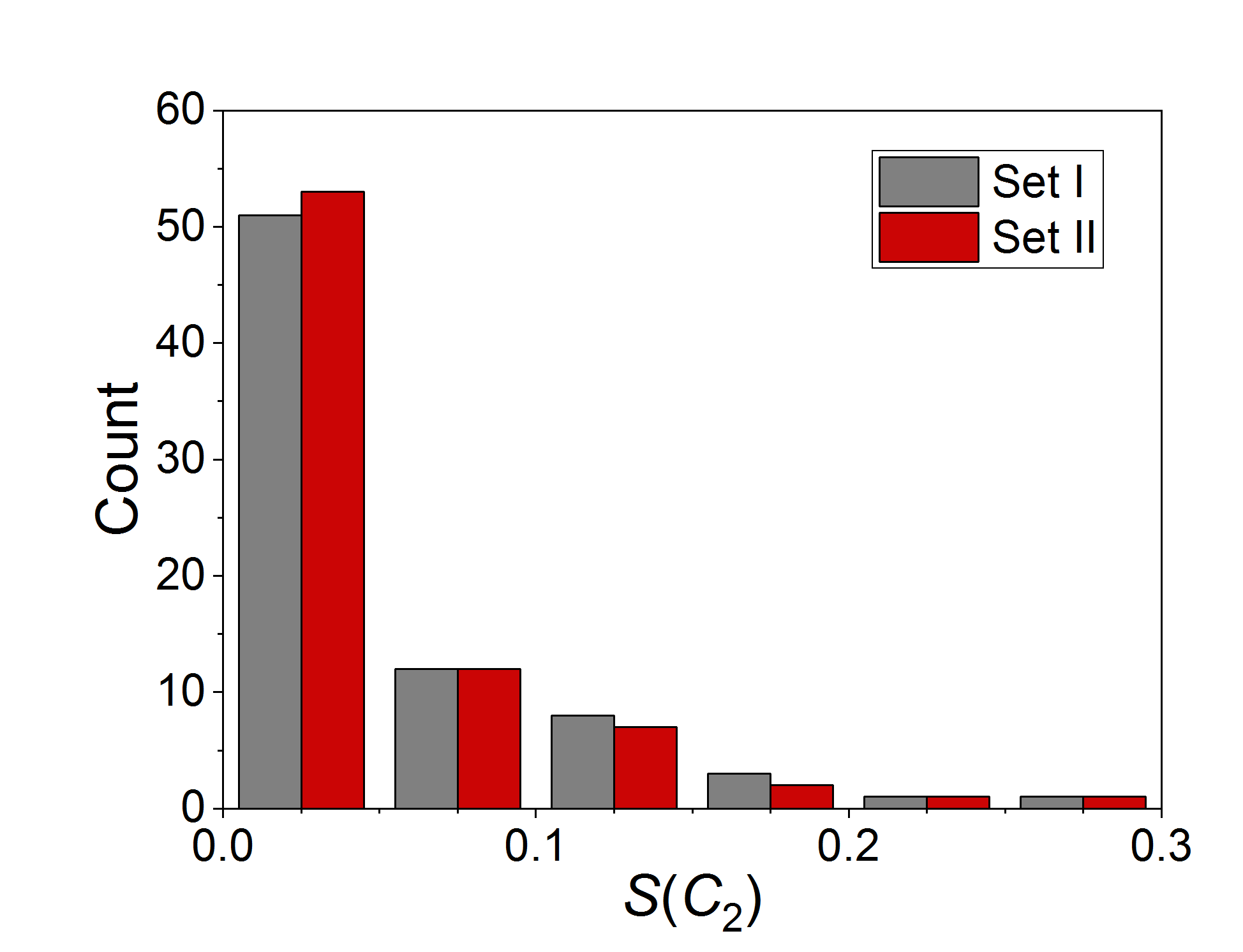

Supplement: S2 Fig — Bin size was set to 0.05. The right tail of the distribution is hidden to increase visibility. (TIF) [file pone.0235863.s003.tif]

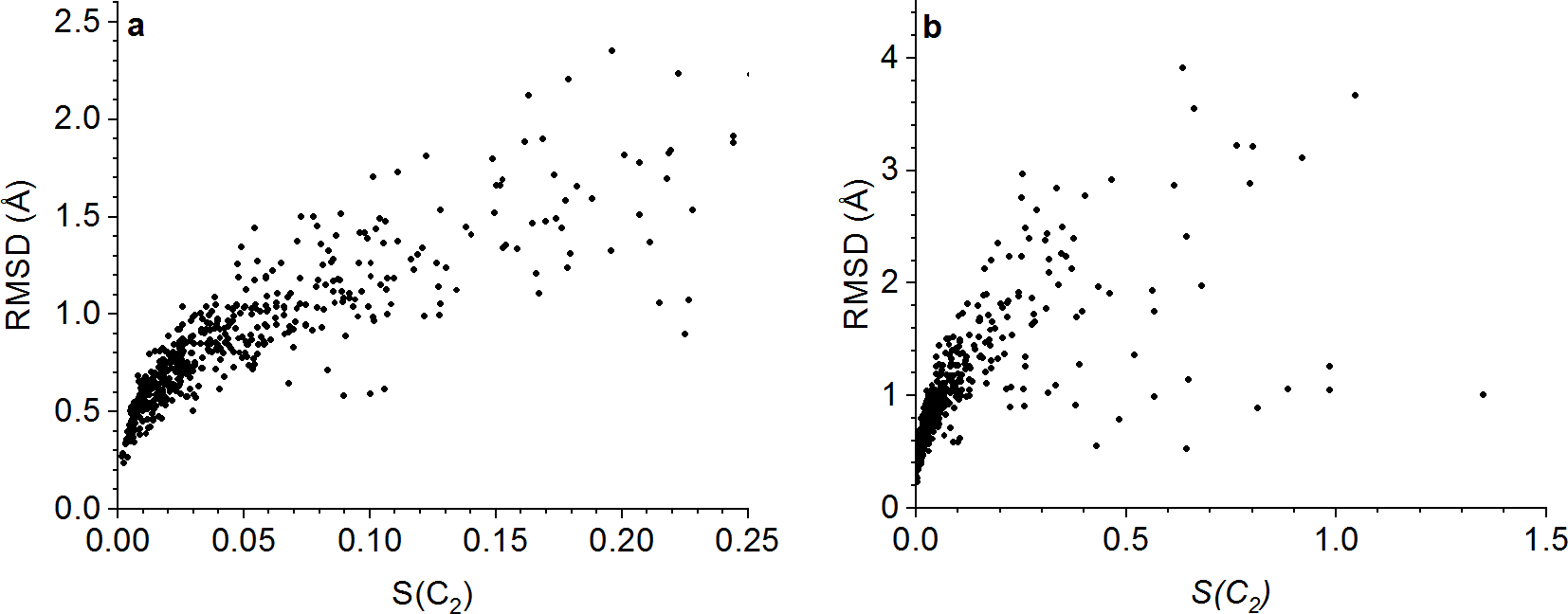

Supplement: S3 Fig — a. ca. 90% of the homodimers in our data set. b. All proteins in the main set. (TIF) [file pone.0235863.s004.tif]
