# Supplementary material for: Side chain flexibility and the symmetry of protein homodimers
Source: PLoS One. 2020 Jul 24;15(7):e0235863. doi: 10.1371/journal.pone.0235863 (PMC7380632; doi:10.1371/journal.pone.0235863)
Supplement: S1 Table — (DOCX) [file pone.0235863.s008.docx]

S1 Table. **Fitting parameters of the log-normal distributions in Fig 3 and S1 Fig.**

| $y=y_{0}+\frac{A}{\sqrt{2\pi}wx}\cdot\exp\left[-\frac{\left[\ln\left(x/xc\right)\right]^{2}}{2w^{2}}\right]$ | **All atoms** | | **Backbone atoms** | |
| --- | --- | --- | --- | --- |
|  | **Value** | **Standard error** | **Value** | **Standard error** |
| **y_0_** | 0.61172 | 0.2025 | 0.16106 | 0.03857 |
| **xc** | 0.03079 | 0.00085 | 0.00898 | 0.00021 |
| **w** | 0.97008 | 0.01713 | 1.25065 | 0.01466 |
| **A** | 4.72178 | 0.08068 | 0.90146 | 0.00949 |
| **Mean** | 0.04929 | 0.00206 | 0.01964 | 0.00079 |
| **Standard deviation** | 0.06162 | 0.00418 | 0.03817 | 0.00239 |
| **R^2^ (coefficient of determination )** | 0.97533 |  | 0.96370 |  |
| **Adjusted  R^2^** | 0.97477 |  | 0.96353 |  |
