# Supplementary material for: Side chain flexibility and the symmetry of protein homodimers
Source: PLoS One. 2020 Jul 24;15(7):e0235863. doi: 10.1371/journal.pone.0235863 (PMC7380632; doi:10.1371/journal.pone.0235863)
Supplement: S2 Table — (DOCX) [file pone.0235863.s009.docx]

**S2 Table. Descriptive statistics of S(*C*_2_) for the double-dimers set.**

| **Attribute** | **Combined set (N=160)** | **Set I:**  **(N = 80)** | **Set II**  **(N = 80)** |
| --- | --- | --- | --- |
| **Mean** | 0.0945 | 0.1052 | 0.08385 |
| **Standard deviation** | 0.2100 | 0.2495 | 0.1621 |
| **SE of mean** | 0.0166 | 0.0279 | 0.0181 |
| **Minimum** | 0.0043 | 0.0043 | 0.0060 |
| **Median** | 0.0307 | 0.0307 | 0.0308 |
| **Maximum** | 1.4325 | 1.4325 | 0.9956 |
