# Supplementary material for: Side chain flexibility and the symmetry of protein homodimers
Source: PLoS One. 2020 Jul 24;15(7):e0235863. doi: 10.1371/journal.pone.0235863 (PMC7380632; doi:10.1371/journal.pone.0235863)
Supplement: S3 Table — (DOCX) [file pone.0235863.s010.docx]

S3 Table. **Fitting parameters for the exponential decay distributions of Fig 5.**

| $y=A_{1}e^{-x/t_{1}}+y_{0}$ | **All atoms** | | **Backbone atoms** | |
| --- | --- | --- | --- | --- |
|  | **Value** | **Standard error** | **Value** | **Standard error** |
| ***A_1_*** | 33,785.0900 | 333.1394 | 44,640.4682 | 581.0953 |
| ***t_1_*** | 0.1528 | 0.0025 | 0.1312 | 0.0027 |
| ***y_0_*** | 611.4411 | 66.7901 | 394.7367 | 101.7586 |
| **R^2^ (coefficient of determination )** | 0.9984 |  | 0.9974 |  |
| **Adjusted  R^2^** | 0.9983 |  | 0.9972 |  |
