# Supplementary material for: Side chain flexibility and the symmetry of protein homodimers
Source: PLoS One. 2020 Jul 24;15(7):e0235863. doi: 10.1371/journal.pone.0235863 (PMC7380632; doi:10.1371/journal.pone.0235863)
Supplement: S4 Table — (DOCX) [file pone.0235863.s011.docx]

S4 Table. **Mismatch of secondary-structure annotation.**

| **Secondary structure** | **Number of residue pairs with equal annotation** | **Total number of residue pairs** | **% mismatch** |
| --- | --- | --- | --- |
| **E – Extended strand, participating in β ladder** | 27,626 | 27,864 | 0.9% |
| **H – α-Helix** | 43,710 | 44,236 | 1.2% |
| **C – Coil** | 23,040 | 23,831 | 3.3% |
| **T – Hydrogen bond turn** | 14,019 | 14,851 | 5.6% |
| **B – Residue in isolated β-bridge** | 1,452 | 1,540 | 5.7% |
| **S – Bend** | 10,044 | 10,776 | 6.8% |
| **G – 3-Helix (3_10_ helix)** | 5,023 | 5,398 | 6.9% |
| **I – 5-Helix (π-helix)** | 848 | 915 | 7.3% |
| **Total** | 125,762 | 129,411 | 2.8% |
