# Supplementary material for: Side chain flexibility and the symmetry of protein homodimers
Source: PLoS One. 2020 Jul 24;15(7):e0235863. doi: 10.1371/journal.pone.0235863 (PMC7380632; doi:10.1371/journal.pone.0235863)
Supplement: S5 Table — Counts represent number of matching residue pairs. Percentages are relative to the subset. (DOCX) [file pone.0235863.s012.docx]

S5 Table. **Amino acid abundance in the subset of the 10% most distorted pairs, of the main set, compared to the general population.** Counts represent number of matching residue pairs. Percentages are relative to the subset.

| **Amino**  **acid** | **Top 10%**  ***d*_i_ > 0.96**  **(N = 12,941)** | | **General population**  **(N = 129,411)** | | **Abundance**  **ratio** |
| --- | --- | --- | --- | --- | --- |
|  | **Count** | **%** | **Count** | **%** |  |
| **Lys** | 1,983 | 15.32 | 6,638 | 5.13 | 2.99 |
| **Gln** | 1,057 | 8.17 | 4,675 | 3.61 | 2.26 |
| **Glu** | 1,833 | 14.16 | 8,181 | 6.32 | 2.24 |
| **Arg** | 1,145 | 8.85 | 6,395 | 4.94 | 1.79 |
| **Asn** | 931 | 7.19 | 5,248 | 4.06 | 1.77 |
| **Met** | 364 | 2.81 | 2,477 | 1.91 | 1.47 |
| **Pro** | 567 | 4.38 | 6,142 | 4.75 | 1.34 |
| **Asp** | 1,032 | 7.97 | 7,704 | 5.95 | 1.07 |
| **Ile** | 509 | 3.93 | 7,423 | 5.74 | 0.92 |
| **Ser** | 778 | 6.01 | 7,260 | 5.61 | 0.90 |
| **Thr** | 627 | 4.85 | 6,970 | 5.39 | 0.68 |
| **His** | 193 | 1.49 | 3,214 | 2.48 | 0.60 |
| **Cys** | 54 | 0.42 | 1,496 | 1.16 | 0.53 |
| **Leu** | 622 | 4.81 | 11,766 | 9.09 | 0.48 |
| **Val** | 457 | 3.53 | 9,481 | 7.33 | 0.36 |
| **Gly** | 218 | 1.68 | 10,421 | 8.05 | 0.25 |
| **Ala** | 292 | 2.26 | 12,253 | 9.47 | 0.24 |
| **Trp** | 39 | 0.30 | 1,893 | 1.46 | 0.24 |
| **Phe** | 135 | 1.04 | 5,386 | 4.16 | 0.21 |
| **Tyr** | 105 | 0.81 | 4,388 | 3.39 | 0.21 |
