# Supplementary material for: Side chain flexibility and the symmetry of protein homodimers
Source: PLoS One. 2020 Jul 24;15(7):e0235863. doi: 10.1371/journal.pone.0235863 (PMC7380632; doi:10.1371/journal.pone.0235863)
Supplement: S6 Table — Counts represent number of matching residue pairs. Percentages are relative to the subset. (DOCX) [file pone.0235863.s013.docx]

Table S6. **Amino acid abundance in the subset of the 10% most distorted pairs compared to the general population of the first subset of double-dimers.** Counts represent number of matching residue pairs. Percentages are relative to the subset.

| **Amino Acid** | **Top 10%**  **d_i_>0.96**  **(N=1,968)** | | **General Population (N=19,679)** | | **Abundance Ratio** |
| --- | --- | --- | --- | --- | --- |
|  | **Count** | **%** | **Count** | **%** |  |
| Lys | 323 | 16.41% | 1,045 | 5.31% | 3.09 |
| Gln | 179 | 9.10% | 721 | 3.66% | 2.48 |
| Glu | 279 | 14.18% | 1,238 | 6.29% | 2.25 |
| Asn | 141 | 7.16% | 824 | 4.19% | 1.71 |
| Arg | 142 | 7.22% | 908 | 4.61% | 1.56 |
| Met | 60 | 3.05% | 411 | 2.09% | 1.46 |
| Asp | 162 | 8.23% | 1,165 | 5.92% | 1.39 |
| Ser | 111 | 5.64% | 1,030 | 5.23% | 1.08 |
| Pro | 91 | 4.62% | 946 | 4.81% | 0.96 |
| Thr | 103 | 5.23% | 1,179 | 5.99% | 0.87 |
| Ile | 83 | 4.22% | 1,179 | 5.99% | 0.70 |
| His | 34 | 1.73% | 517 | 2.63% | 0.66 |
| Leu | 89 | 4.52% | 1,722 | 8.75% | 0.52 |
| Val | 66 | 3.35% | 1,409 | 7.16% | 0.47 |
| Ala | 38 | 1.93% | 1,788 | 9.09% | 0.21 |
| Phe | 15 | 0.76% | 765 | 3.89% | 0.20 |
| Gly | 32 | 1.63% | 1,657 | 8.42% | 0.19 |
| Cys | 5 | 0.25% | 259 | 1.32% | 0.19 |
| Tyr | 11 | 0.56% | 631 | 3.21% | 0.17 |
| Trp | 4 | 0.20% | 285 | 1.45% | 0.14 |
