# Supplementary material for: Side chain flexibility and the symmetry of protein homodimers
Source: PLoS One. 2020 Jul 24;15(7):e0235863. doi: 10.1371/journal.pone.0235863 (PMC7380632; doi:10.1371/journal.pone.0235863)
Supplement: S7 Table — Counts represent number of matching residue pairs. Percentages are relative to the subset. (DOCX) [file pone.0235863.s014.docx]

Table S7. **Amino acid abundance in the subset of the 10% most distorted pairs compared to the general population of the second subset of double-dimers.** Counts represent number of matching residue pairs. Percentages are relative to the subset.

| **Amino Acid** | **Top 10%**  **d_i_>0.96**  **(N=1,968)** | | **General Population (N=19,679)** | | **Abundance Ratio** |
| --- | --- | --- | --- | --- | --- |
|  | **Count** | **%** | **Count** | **%** |  |
| Lys | 317 | 16.11% | 1,045 | 5.31% | 3.03 |
| Gln | 184 | 9.35% | 721 | 3.66% | 2.55 |
| Glu | 261 | 13.26% | 1,238 | 6.29% | 2.11 |
| Asn | 137 | 6.96% | 824 | 4.19% | 1.66 |
| Arg | 144 | 7.32% | 908 | 4.61% | 1.59 |
| Met | 62 | 3.15% | 411 | 2.09% | 1.51 |
| Asp | 163 | 8.28% | 1,165 | 5.92% | 1.40 |
| Ser | 114 | 5.79% | 1,030 | 5.23% | 1.11 |
| Thr | 104 | 5.28% | 1,179 | 5.99% | 0.88 |
| Pro | 74 | 3.76% | 946 | 4.81% | 0.78 |
| Ile | 86 | 4.37% | 1,179 | 5.99% | 0.73 |
| His | 30 | 1.52% | 517 | 2.63% | 0.58 |
| Val | 75 | 3.81% | 1,409 | 7.16% | 0.53 |
| Leu | 90 | 4.57% | 1,722 | 8.75% | 0.52 |
| Phe | 22 | 1.12% | 765 | 3.89% | 0.29 |
| Ala | 49 | 2.49% | 1,788 | 9.09% | 0.27 |
| Cys | 7 | 0.36% | 259 | 1.32% | 0.27 |
| Tyr | 14 | 0.71% | 631 | 3.21% | 0.22 |
| Gly | 33 | 1.68% | 1,657 | 8.42% | 0.20 |
| Trp | 2 | 0.10% | 285 | 1.45% | 0.07 |
